# Supplementary material for: Whole genome sequence analysis of blood lipid levels in >66,000 individuals
Source: Nat Commun. 2022 Oct 11;13:5995. doi: 10.1038/s41467-022-33510-7 (PMC9553944; doi:10.1038/s41467-022-33510-7)
Supplement: Supplementary file 1 — Supplementary Information [file 41467_2022_33510_MOESM1_ESM.pdf]

Supplementary Note1:

Old Order Amish (Amish, n=1,083):
TOPMed dbGaP accession#: phs000956, Parent dbGaP accession#: phs000391, Sequencing Center: Broad Institute of MIT and Harvard.
The Old Order Amish (OOA) population of Lancaster County, PA immigrated to the Colonies from Western Europe in the early 1700's. Investigators at University of Maryland School of Medicine have been studying the genetic determinants of cardiometabolic health in this population since 1993. To date, over 7,000 Amish adults have participated in one or more of our studies. The Heredity and Phenotype Intervention (HAPI) Heart Study was initiated in 2002 and chose to study the OOA to test the genetic effects of complex phenotypes from this genetically homogeneous population[1]. The Amish Research Group includes investigators with a diverse range of interests in population and basic science and in clinical and translational research. Whole genome sequencing (WGS) for the Trans-Omics in Precision Medicine (TOPMed) program was supported by the National Heart, Lung and Blood Institute (NHLBI). The Amish studies were supported by NIH grants R01 AG18728, U01 HL072515, R01 HL088119, R01 HL121007, and P30 DK072488.

Atherosclerosis Risk in Communities study (ARIC, n=8,016):
TOPMed dbGaP accession#: phs001211, Parent dbGaP accession#: phs000280, Sequencing Center: Baylor College of Medicine Human Genome Sequencing Center and Broad Institute of MIT and Harvard.
The ARIC study is a population-based prospective cohort study of cardiovascular disease sponsored by the National Heart, Lung, and Blood Institute (NHLBI). ARIC included 15,792 individuals, predominantly European American and African American, aged 45-64 years at baseline (1987-89), chosen by probability sampling from four US communities. Cohort members completed three additional triennial follow-up examinations, a fifth exam in 2011-2013, a sixth exam in 2016-2017, a seventh exam in 2018-2019, and an eight exam in 2020. The ARIC study has been described in detail previously[2].
Whole genome sequencing (WGS) for the Trans-Omics in Precision Medicine (TOPMed) program was supported by the National Heart, Lung and Blood Institute (NHLBI). WGS for "NHLBI TOPMed: Atherosclerosis Risk in Communities (ARIC)" (phs001211) was performed at the Baylor College of Medicine Human Genome Sequencing Center (HHSN268201500015C and 3U54HG003273-12S2) and the Broad Institute for MIT and Harvard (3R01HL092577-06S1). Centralized read mapping and genotype calling, along with variant quality metrics and filtering were provided by the TOPMed Informatics Research Center (3R01HL-117626-02S1). Phenotype harmonization, data management, sample-identity QC, and general study coordination, were provided by the TOPMed Data Coordinating Center (3R01HL-120393-02S1). We gratefully acknowledge the studies and participants who provided biological samples and data for TOPMed.
The Genome Sequencing Program (GSP) was funded by the National Human Genome Research Institute (NHGRI), the National Heart, Lung, and Blood Institute (NHLBI), and

the National Eye Institute (NEI). The GSP Coordinating Center (U24 HG008956) contributed to cross-program scientific initiatives and provided logistical and general study coordination. The Centers for Common Disease Genomics (CCDG) program was supported by NHGRI and NHLBI, and whole genome sequencing was performed at the Baylor College of Medicine Human Genome Sequencing Center (UM1 HG008898 and R01HL059367).
The Atherosclerosis Risk in Communities study has been funded in whole or in part with Federal funds from the National Heart, Lung, and Blood Institute, National Institutes of Health, Department of Health and Human Services (contract numbers HHSN268201700001I, HHSN268201700002I, HHSN268201700003I, HHSN268201700004I and HHSN268201700005I). The authors thank the staff and participants of the ARIC study for their important contributions.

Mt Sinai BioMe Biobank (BioMe, n=9,848):
TOPMed dbGaP accession#: phs001644, Parent dbGaP accession#: phs000925, Sequencing Center: Baylor College of Medicine Human Genome Sequencing Center, Northwest Genomics Center
The Institute for Personalized Medicine at the Icahn School of Medicine at Mount Sinai is leading the movement toward diagnosis and classification of disease according to the patient's molecular profile. BioMe is the major effort towards this goal, BioMe, an electronic medical record-linked biobank that enables researchers to conduct genetic, epidemiologic, molecular, and genomic studies rapidly and efficiently on large collections of research specimens linked with medical information. BioMed cohort is composed of White, Black, Hispanic and Asian populations. The Mount Sinai Medical Center services diverse local communities of upper Manhattan, including Central Harlem (86% African American), East Harlem (88% Hispanic Latino), and Upper East Side (88% Caucasian/white) with broad health disparities[3]. Whole genome sequencing (WGS) for the Trans-Omics in Precision Medicine (TOPMed) program was supported by the National Heart, Lung and Blood Institute (NHLBI). The Mount Sinai BioMe Biobank has been supported by The Andrea and Charles Bronfman Philanthropies and in part by Federal funds from the NHLBI and NHGRI (U01HG00638001; U01HG007417; X01HL134588).

Coronary Artery Risk Development in Young Adults (CARDIA, n=3,056):
TOPMed dbGaP accession#: *phs001612*, Parent dbGaP accession#: *phs000285*, *Sequencing Center: Baylor College of Medicine Human Genome Sequencing Center.*
The Coronary Artery Risk Development in Young Adults (CARDIA) Study is a study examining the development and determinants of clinical and subclinical cardiovascular disease and their risk factors. It began in 1985-6 with a group of 5115 black and white men and women aged 18-30 years[4]. The participants were selected so that there would be approximately the same number of people in subgroups of race, gender, education (high school or less and more than high school) and age (18-24 and 25-30) in each of 4 centers: Birmingham, AL; Chicago, IL; Minneapolis, MN; and Oakland, CA. These same participants were asked to participate in follow-up examinations during 1987-1988 (Year 2), 1990-1991 (Year 5), 1992-1993 (Year 7), 1995-1996 (Year 10), 2000-2001 (Year 15), 2005-2006 (Year 20), 2010-2011 (Year 25), and 2015-2016 (Year 30).

CARDIA cohort is composed of White and Black populations. Whole genome sequencing (WGS) for the Trans-Omics in Precision Medicine (TOPMed) program was supported by the National Heart, Lung and Blood Institute (NHLBI). The Coronary Artery Risk Development in Young Adults Study (CARDIA) is conducted and supported by the National Heart, Lung, and Blood Institute (NHLBI) in collaboration with the University of Alabama at Birmingham (HHSN268201800005I & HHSN268201800007I), Northwestern University (HHSN268201800003I), University of Minnesota (HHSN268201800006I), and Kaiser Foundation Research Institute (HHSN268201800004I). CARDIA was also partially supported by the Intramural Research Program of the National Institute on Aging (NIA) and an intra-agency agreement between NIA and NHLBI (AG0005).

Cleveland Family Study (CFS, n=579):
TOPMed dbGaP accession#: phs000954, Parent dbGaP accession#: phs000284, Sequencing Center: McDonnell Genome Institute (MGI) at Washington University.
The CFS is a family-based longitudinal study that includes participants with laboratory diagnosed sleep apnea, their family members and neighborhood control families followed between 1990 and 2006[5]. After an overnight fast, blood was collected which was assayed for lipid levels at the University of Vermont Laboratory for Clinical Biochemistry Research. Lipids (triglycerides, HDL cholesterol) from fasted blood serum were measured by enzymatic methods. CFS cohort is composed of White and Black populations. Whole genome sequencing (WGS) for the Trans-Omics in Precision Medicine (TOPMed) program was supported by the National Heart, Lung and Blood Institute (NHLBI). CFS is supported by grants from the NHLBI (HL046389, HL113338, and 1R35HL135818).

Cardiovascular Health Study (CHS, n=3,456):
TOPMed dbGaP accession#: phs001368, Parent dbGaP accession#: phs000287, Sequencing Center: Baylor College of Medicine Human Genome Sequencing Center.
The Cardiovascular Health Study (CHS) is an NHLBI-funded observational study of risk factors for cardiovascular disease in adults 65 years or older. Starting in 1989, and continuing through 1999, participants underwent annual extensive clinical examinations. Measurements included traditional risk factors such as blood pressure and lipids. Additionally, measures of subclinical disease, including echocardiography of the heart, carotid ultrasound, and cranial magnetic-resonance imaging (MRI) were determined. At six-month intervals between clinic visits, and once clinic visits ended, participants were contacted by phone to ascertain hospitalizations and health status. The main outcomes are coronary heart disease (CHD), angina, heart failure (HF), stroke, transient ischemic attack (TIA), claudication, and mortality[6]. Participants continue to be contacted by phone every 6 months. Participants from four counties were included in the study: Forsyth County, North Carolina; Sacramento County, California; Washington County, Maryland; and Pittsburgh, Pennsylvania. CHS cohort is composed of White and Black populations. Whole genome sequencing (WGS) for the Trans-Omics in Precision Medicine (TOPMed) program was supported by the National Heart, Lung and Blood Institute (NHLBI). The CHS research was supported by NHLBI contracts 75N92021D00006, HHSN268201200036C, HHSN268200800007C, HHSN268201800001C, N01HC55222, N01HC85079, N01HC85080, N01HC85081,

N01HC85082, N01HC85083, N01HC85086; and NHLBI grants U01HL080295, R01HL087652, R01HL105756, R01HL103612, R01HL120393, R01HL130114, and R01 HL059367, with additional contribution from the National Institute of Neurological Disorders and Stroke (NINDS). Additional support was provided through R01AG023629 from the National Institute on Aging (NIA).

Diabetes Heart Study (DHS, n=365):
TOPMed dbGaP accession#: phs001412, Parent dbGaP accession#: phs001012, Sequencing Center: Broad Institute of MIT and Harvard
The Diabetes Heart Study (DHS) is a family-based study enriched for type 2 diabetes (T2D). The cohort was recruited between 1998 and 2006. Participants were extensively phenotyped for measures of subclinical CVD and other known CVD risk factors[7]. Primary outcomes were quantified burden of vascular calcified plaque in the coronary artery, carotid artery, and abdominal aorta all determined from non-contrast computed tomography scans. Whole genome sequencing (WGS) for the Trans-Omics in Precision Medicine (TOPMed) program was supported by the National Heart, Lung and Blood Institute (NHLBI). The DHS research was supported by R01 HL92301, R01 HL67348, R01 NS058700, R01 AR48797, R01 DK071891, R01 AG058921, the General Clinical Research Center of the Wake Forest University School of Medicine (M01 RR07122, F32 HL085989), the American Diabetes Association, and a pilot grant from the Claude Pepper Older Americans Independence Center of Wake Forest University Health Sciences (P60 AG10484).

Framingham Heart Study (FHS, n=3,992):
TOPMed dbGaP accession#: phs000974, Parent dbGaP accession#: phs000007, Sequencing Center: Broad Institute of MIT and Harvard

The Framingham Heart Study (FHS) is a prospective cohort study of 3 generations of subjects who have been followed up to 65 years to evaluate risk factors for cardiovascular disease.[13-16] Its large sample of ~15,000 men and women who have been extensively phenotyped with repeated examinations make it ideal for the study of genetic associations with cardiovascular disease risk factors and outcomes. DNA samples have been collected and immortalized since the mid-1990s and are available on ~8000 study participants in 1037 families. These samples have been used for collection of GWAS array data and exome chip data in nearly all with DNA samples, and for targeted sequencing, deep exome sequencing and light coverage whole genome sequencing in limited numbers. Additionally, mRNA and miRNA expression data, DNA methylation data, metabolomics and other 'omics data are available on a sizable portion of study participants. This project on the focuses on deep whole genome sequencing (mean 30X coverage) in 3,992 subjects with lipid data available.

FHS acknowledges the support of contracts NO1-HC-25195 and HHSN268201500001I from the National Heart, Lung and Blood Institute and grant supplement R01 HL092577-06S1 for this research. WGS for “NHLBI TOPMed: Whole Genome Sequencing and Related Phenotypes in the Framingham Heart Study” (phs000974) was performed at the Broad Institute of MIT and Harvard (HHSN268201500014C, 3R01HL092577-06S1, and 3U54HG003067-12S2). We also

acknowledge the dedication of the FHS study participants without whom this research would not be possible.

Genetic Studies of Atherosclerosis Risk (GeneSTAR, n=1,757):
TOPMed dbGaP accession#: phs001218, Parent dbGaP accession#: phs000375, Sequencing Center: Broad Institute of MIT and Harvard, Illumina Genomic Services, PSOMAGEN (formerly Macrogen).
GeneSTAR is a family-based study in initially healthy brothers and sisters, and offspring of people with early-onset coronary disease. The goal is to discover and amplify mechanisms of stroke and coronary heart disease. In 1982 GeneSTAR was created to study patterns of coronary heart disease and related risk factors in families with early-onset coronary disease, identified from10 Baltimore area Hospitals. Extensive additional cardiovascular testing and risk assessment was done at baseline and serially[12,13]. Follow-up was carried out to determine incident cardiovascular disease, stroke, peripheral arterial disease, diabetes, cancer, and related comorbidities, from 5 to 30 years after study entry. Whole genome sequencing (WGS) for the Trans-Omics in Precision Medicine (TOPMed) program was supported by the National Heart, Lung and Blood Institute (NHLBI). GeneSTAR was supported by grants from the National Institutes of Health/National Heart, Lung, and Blood Institute (U01 HL72518, HL087698, HL49762, HL58625, HL071025, HL112064), the National Institutes of Health/National Institute of Nursing Research (NR0224103), and by a grant from the National Institutes of Health/National Center for Research Resources (M01-RR000052) to the Johns Hopkins General Clinical Research Center.

Genetic Epidemiology Network of Arteriopathy (GENOA, n=1,046):
TOPMed dbGaP accession#: phs001345, Parent dbGaP accession#: phs001238, Sequencing Center: Broad Institute of MIT and Harvard, McDonnell Genome Institute (MGI) at Washington University.
The Genetic Epidemiology Network of Arteriopathy (GENOA) is one of four networks in the NHLBI Family-Blood Pressure Program (FBPP)[14]. GENOA's long-term objective is to elucidate the genetics of target organ complications of hypertension, including both atherosclerotic and arteriolosclerotic complications involving the heart, brain, kidneys, and peripheral arteries[15]. The longitudinal GENOA Study recruited European-American and African-American sibships with at least 2 individuals with clinically diagnosed essential hypertension before age 60 years. All other members of the sibship were invited to participate regardless of their hypertension status. Participants were diagnosed with hypertension if they had either 1) a previous clinical diagnosis of hypertension by a physician with current anti-hypertensive treatment, or 2) an average systolic blood pressure ≥ 140 mm Hg or diastolic blood pressure ≥ 90 mm Hg based on the second and third readings at the time of their clinic visit. Only participants of the African-American Cohort were sequenced through TOPMed. Whole genome sequencing (WGS) for the Trans-Omics in Precision Medicine (TOPMed) program was supported by the National Heart, Lung and Blood Institute (NHLBI). Support for GENOA was provided by the National Heart, Lung and Blood Institute (HL054457, HL054464, HL054481, and HL087660) of the National Institutes of Health.

Genetic Epidemiology Network of Salt Sensitivity (GenSalt, n=1,772):
TOPMed dbGaP accession#: phs001217, Parent dbGaP accession#: phs000784, Sequencing Center: Broad Institute of MIT and Harvard.
GenSalt utilizes a family feeding-study design. Each family is ascertained through a proband with untreated prehypertension or stage-1 hypertension in rural China. Medical history, lifestyle risk factors, and cold pressor tests are obtained at baseline visits while BP, weight, blood and urine specimens are collected at baseline and follow-up visits[16]. The dietary intervention includes a 7-day low sodium-feeding (51.3 mmol/day), a 7-day high sodium-feeding (307.8 mmol/day), and a 7-day high sodium-feeding with an oral potassium supplementation (60 mmol/day). Whole genome sequencing (WGS) for the Trans-Omics in Precision Medicine (TOPMed) program was supported by the National Heart, Lung and Blood Institute (NHLBI). GenSalt was supported by research grants (U01HL072507, R01HL087263, and R01HL090682) from the National Heart, Lung and Blood Institute, National Institutes of Health, Bethesda, MD.

Genetics of Lipid-Lowering Drugs and Diet Network (GOLDN, n=926):
TOPMed dbGaP accession#: phs001359, Parent dbGaP accession#: phs000741, Sequencing Center: McDonnell Genome Institute (MGI) at Washington University.
GOLDN is a family-based study of European descent individuals recruited in Minneapolis and Salt Lake City (two of the NHLBI Family Heart Study sites). Contributions of genes, shared and individual environments, and behaviors to variations in risk factors, preclinical atherosclerosis, and CHD were estimated in the study[17]. It aims to uncover genetic predictors of variability in lipid phenotypes, which include both fasting and postprandial lipids quantified using traditional methods, NMR, and high throughput lipidomics. Whole genome sequencing (WGS) for the Trans-Omics in Precision Medicine (TOPMed) program was supported by the National Heart, Lung and Blood Institute (NHLBI). GOLDN biospecimens, baseline phenotype data, and intervention phenotype data were collected with funding from National Heart, Lung and Blood Institute (NHLBI) grant U01 HL072524.

Hispanic Community Health Study - Study of Latinos (HCHS_SOL, n=7714):
TOPMed dbGaP accession#: phs001395, Parent dbGaP accession#: phs000810, Sequencing Center: Baylor College of Medicine Human Genome Sequencing Center.
The Hispanic Community Health Study (HCHS)/Study of Latinos (SOL) is a multicenter, community-based cohort study of Hispanic/Latino adults in the United States. The main goal of the study is to identify risk factors which could either be protective or harmful to the Hispanic community[18,19]. A total of 16,000 Hispanic/Latino individuals of age 18-74 were recruited. Participants are recruited in community areas surrounding four field centers in the Bronx, Chicago, Miami, and San Diego. Whole genome sequencing (WGS) for the Trans-Omics in Precision Medicine (TOPMed) program was supported by the National Heart, Lung and Blood Institute (NHLBI). The Hispanic Community Health Study/Study of Latinos was carried out as a collaborative study supported by contracts from the National Heart, Lung, and Blood Institute (NHLBI) to the University of North Carolina (N01-HC65233), University of Miami (N01-HC65234), Albert Einstein College of Medicine (N01-HC65235), Northwestern University (N01-HC65236), and San Diego State University (N01-HC65237).

Hypertension Genetic Epidemiology Network and Genetic Epidemiology Network of Arteriopathy (HyperGEN, n=1,853):
TOPMed dbGaP accession#: phs001293, Parent dbGaP accession#: phs001293, Sequencing Center: McDonnell Genome Institute (MGI) at Washington University.
The Hypertension Genetic Epidemiology Network Study (HyperGEN) - Genetics of Left Ventricular (LV) Hypertrophy is a familial study aimed to understand genetic risk factors for LV hypertrophy by conducting genetic studies of continuous traits from echocardiography exams [20,21]. As part of HyperGEN study, four field centers recruited African American and white hypertensive siblings, aged 23 to 87 years. Data from detailed clinical exams as well as genotyping data for linkage studies, candidate gene studies and GWAS have been collected and is shared between HyperGEN and the ancillary HyperGEN - Genetics of LV Hypertrophy study. Whole genome sequencing (WGS) for the Trans-Omics in Precision Medicine (TOPMed) program was supported by the National Heart, Lung and Blood Institute (NHLBI). The HyperGEN Study is part of the National Heart, Lung, and Blood Institute (NHLBI) Family Blood Pressure Program; collection of the data represented here was supported by grants U01 HL054472 (MN Lab), U01 HL054473 (DCC), U01 HL054495 (AL FC), and U01 HL054509 (NC FC). The HyperGEN: Genetics of Left Ventricular Hypertrophy Study was supported by NHLBI grant R01 HL055673 with whole-genome sequencing made possible by supplement -18S1.

Jackson Heart Study (JHS, n=2,847):
TOPMed dbGaP accession#: phs000964, Parent dbGaP accession#: phs000286, Sequencing Center: McDonnell Genome Institute (MGI) at Washington University.
The JHS is a large, community-based, observational study, four subsamples of participants (random, volunteer, ARIC (continuing from Atherosclerosis Risk in Communities study), and family) residing in the Jackson, Mississippi metropolitan statistical area (MSA) were included[22,23,24]. Recruitment was limited to persons 35-84 years old except in the family cohort, where those 21 years old and above were eligible. Participants provided extensive medical and social history, had an array of physical and biochemical measurements and diagnostic procedures, and provided genomic DNA. Whole genome sequencing (WGS) for the Trans-Omics in Precision Medicine (TOPMed) program was supported by the National Heart, Lung and Blood Institute (NHLBI). The Jackson Heart Study (JHS) is supported and conducted in collaboration with Jackson State University (HHSN268201800013I), Tougaloo College (HHSN268201800014I), the Mississippi State Department of Health (HHSN268201800015I) and the University of Mississippi Medical Center (HHSN268201800010I, HHSN268201800011I and HHSN268201800012I) contracts from the National Heart, Lung, and Blood Institute (NHLBI) and the National Institute on Minority Health and Health Disparities (NIMHD).

Multi-Ethnic Study of Atherosclerosis (MESA, n=5,290):
TOPMed dbGaP accession#: phs001416, Parent dbGaP accession#: phs000209, Sequencing Center: Broad Institute of MIT and Harvard.

The Multi-Ethnic Study of Atherosclerosis (MESA) is a study of the characteristics of subclinical cardiovascular disease (disease detected non-invasively before it has produced clinical signs and symptoms) and the risk factors that predict progression to clinically overt cardiovascular disease or progression of the subclinical disease. MESA researchers study a diverse, population-based sample of 6,814 asymptomatic men and women aged 45-84 from six field centers across the United States. Approximately 38 % of the recruited participants are white, 28 % African-American, 22 % Hispanic, and 12 % Asian, predominantly of Chinese descent[25]. Six exams have been completed since 2000. Participants are contacted every 9 to 12 months throughout the study to assess clinical morbidity and mortality. The final 18 months of the study will be dedicated to close out and data analysis and publication. Baseline measurements will include measurement of coronary calcium using computed tomography; measurement of ventricular mass and function using cardiac magnetic resonance imaging; measurement of flow-mediated brachial artery endothelial vasodilation, carotid intimal-medial wall thickness, and distensibility of the carotid arteries using ultrasonography; measurement of peripheral vascular disease using ankle and brachial blood pressures; electrocardiography; and assessments of microalbuminuria, standard CVD risk factors, sociodemographic factors, life habits, and psychosocial factors.
Centralized read mapping and genotype calling, along with variant quality metrics and filtering were provided by the TOPMed Informatics Research Center (3R01HL-117626-02S1). Phenotype harmonization, data management, sample-identity QC, and general study coordination, were provided by the TOPMed Data Coordinating Center (3R01HL-120393-02S1). MESA and the MESA SHARe project are conducted and supported by the National Heart, Lung, and Blood Institute (NHLBI) in collaboration with MESA investigators. Whole genome sequencing (WGS) for the Trans-Omics in Precision Medicine (TOPMed) program was supported by the National Heart, Lung and Blood Institute (NHLBI). Support for MESA is provided by contracts HHSN268201500003I, N01-HC-95159, N01-HC-95160, N01-HC-95161, N01-HC-95162, N01-HC-95163, N01-HC-95164, N01-HC-95165, N01-HC-95166, N01-HC-95167, N01-HC-95168, N01-HC-95169, UL1-TR-000040, UL1-TR-001079, UL1-TR-001420. MESA Family is conducted and supported by the National Heart, Lung, and Blood Institute (NHLBI) in collaboration with MESA investigators. Support is provided by grants and contracts R01HL071051, R01HL071205, R01HL071250, R01HL071251, R01HL071258, R01HL071259, and by the National Center for Research Resources, Grant UL1RR033176. The provision of genotyping data was supported in part by the National Center for Advancing Translational Sciences, CTSI grant UL1TR001881, and the National Institute of Diabetes and Digestive and Kidney Disease Diabetes Research Center (DRC) grant DK063491 to the Southern California Diabetes Endocrinology Research Center.

Massachusetts General Hospital Atrial Fibrillation Study (MGH_AF, n=683):
TOPMed dbGaP accession#: phs001062, Parent dbGaP accession#: phs001001
Sequencing Center: Broad Institute of MIT and Harvard.
The MGH-AF study was initiated to study the effects of atrial fibrillations by comparing unaffected and affected family members[26,27]. The participants provide details on past medical history, AF treatment and family history. An electrocardiogram is performed; the results of an echocardiogram are obtained along with blood samples. For the TOPMed

whole genome sequencing project only early-onset atrial fibrillation cases were sequenced. Early-onset atrial fibrillation was defined as an age of onset prior to 66 years of age. Whole genome sequencing (WGS) for the Trans-Omics in Precision Medicine (TOPMed) program was supported by the National Heart, Lung and Blood Institute (NHLBI). The MGH AF Study was supported by grants to Dr. Ellinor from the Fondation Leducq (14CVD01), the National Institutes of Health to Dr. Ellinor (1RO1HL092577, R01HL128914, K24HL105780) and Dr. Lubitz (1R01HL139731) and by grants from the American Heart Association to Dr. Ellinor (18SFRN34110082) and to Dr. Lubitz (18SFRN34250007).

San Antonio Family Study (SAFS, n=619):
TOPMed dbGaP accession#: phs001215, Parent dbGaP accession#: phs000462, Sequencing Center: Illumina Genomic Services.
The SAFS began in 1991, and included 1,431 individuals in 42 extended families at baseline. Probands were 40- to 60-year-old low-income Mexican Americans selected at random without regard to presence or absence of disease, almost exclusively from Mexican American census tracts in San Antonio, Texas. The major objectives of this study are to identify low frequency or rare variants in and around known common variant signals for CVD, as well as to find novel low frequency or rare variants influencing susceptibility to CVD[28]. Whole genome sequencing (WGS) for the Trans-Omics in Precision Medicine (TOPMed) program was supported by the National Heart, Lung and Blood Institute (NHLBI). Collection of the San Antonio Family Study data was supported in part by National Institutes of Health (NIH) grants R01 HL045522, MH078143, MH078111 and MH083824; and whole genome sequencing of SAFS subjects was supported by U01 DK085524 and R01 HL113323. We are very grateful to the participants of the San Antonio Family Study for their continued involvement in our research programs.

Samoan Adiposity Study (SAS, n=1,182):
TOPMed dbGaP accession#: phs000972, Parent dbGaP accession#: phs000914, Sequencing Center: New York Genome Center and McDonnell Genome Institute (MGI) at Washington University.
The main aim of the Samoan Adiposity Study is to identify risk factors for obesity and cardiometabolic phenotypes among the Samoan population. The recruitment and measurement of the study participants took place from February to July 2010 and thirty-three villages were included in the study[29,30]. The participants reside throughout the independent nation of Samoa, which is experiencing economic development and the nutrition transition. Genotyping was performed with the Affymetrix Genome-Wide Human SNP 6.0 Array using a panel of approximately 900,000 SNPs. Anthropometric, fasting blood biomarkers and detailed dietary, physical activity, health and socio-demographic variables were collected. Whole genome sequencing (WGS) for the Trans-Omics in Precision Medicine (TOPMed) program was supported by the National Heart, Lung and Blood Institute (NHLBI). Data collection was funded by NIH grant R01-HL093093. We thank the Samoan participants of the study and local village authorities. We acknowledge the support of the Samoan Ministry of Health and the Samoa Bureau of Statistics for their support of this research.

Taiwan Study of Hypertension using Rare Variants (THRV, n=1,982):
TOPMed dbGaP accession#: phs001387, Parent dbGaP accession#: phs001387, Sequencing Center: Baylor College of Medicine Human Genome Sequencing Center.
The THRV-TOPMed study consists of three cohorts: The SAPPHIRe Family cohort, TSGH (Tri-Service General Hospital, a hospital-based cohort), and TCVGH (Taichung Veterans General Hospital, another hospital-based cohort), all based in Taiwan[31,32]. 1,271 subjects were previously recruited as part of the NHLBI-sponsored SAPPHIRe Network (which is part of the Family Blood Pressure Program, FBPP). THRV is a collaborative study between Washington University in St. Louis, LA BioMed at Harbor UCLA, University of Texas in Houston, Taichung Veterans General Hospital, Taipei Veterans General Hospital, Tri-Service General Hospital, National Health Research Institutes, National Taiwan University, and Baylor University. THRV is based (substantially) on the parent SAPPHIRe study, along with additional population-based and hospital-based cohorts. Whole genome sequencing (WGS) for the Trans-Omics in Precision Medicine (TOPMed) program was supported by the National Heart, Lung and Blood Institute (NHLBI). The Rare Variants for Hypertension in Taiwan Chinese (THRV) is supported by the National Heart, Lung, and Blood Institute (NHLBI) grant (R01HL111249) and its participation in TOPMed is supported by an NHLBI supplement (R01HL111249-04S1). SAPPHIRe was supported by NHLBI grants (U01HL54527, U01HL54498) and Taiwan funds, and the other cohorts were supported by Taiwan funds.

Women's Health Initiative (WHI, n=8,263):
TOPMed dbGaP accession#: phs001237, Parent dbGaP accession#: phs000200, Sequencing Center: Broad Institute of MIT and Harvard.
The Women's Health Initiative (WHI) is a large study of postmenopausal women's health investigating risk factors for cancer, CVD, age-related fractures and chronic disease. It began in 1993 as a set of randomized controlled clinical trials (CT) and an observational study (OS). Specifically, the CT (n=68,132) included three overlapping components: The Hormone Therapy (HT) Trials (n=27,347), Dietary Modification (DM) Trial (n=48,835), and Calcium and Vitamin D (CaD) Trial (n=36,282). Eligible women could be randomized into as many as all three CTs components. Women who were ineligible or unwilling to join the CT were then invited to join the OS (n=93,676)[33,34]. WHI is a case control study with ~5000 strokes and >2000 CHD samples. Whole genome sequencing (WGS) for the Trans-Omics in Precision Medicine (TOPMed) program was supported by the National Heart, Lung and Blood Institute (NHLBI). The WHI program is funded by the National Heart, Lung, and Blood Institute, National Institutes of Health, U.S. Department of Health and Human Services through contracts HHSN268201600018C, HHSN268201600001C, HHSN268201600002C, HHSN268201600003C, and HHSN268201600004C.

TOPMed Omics Support table:

TOPMed Accession #	TOPMed Project	Parent Study	TOPMed Phase	Omics Center	Omics Support	Omics Type
**phs000956**	Amish	Amish	1	Broad Genomics	3R01HL121007-01S1	WGS
**phs001211**	AFGen	ARIC AFGen	1	Broad Genomics	3R01HL092577-06S1	WGS
**phs001211**	VTE	ARIC	2	Baylor	3U54HG003273-12S2 / HHSN268201500015C	WGS
**phs001644**	AFGen	BioMe AFGen	2.4	MGI	3UM1HG008853-01S2	WGS
**phs001644**	BioMe	BioMe	3	MGI	HHSN268201600037I	WGS
**phs001644**	BioMe	BioMe	3	Baylor	HHSN268201600033I	WGS
**phs001612**	CARDIA	CARDIA	3	Baylor	HHSN268201600033I	WGS
**phs000954**	CFS	CFS	3.5	NWGC	HHSN268201600032I	WGS
**phs000954**	CFS	CFS	1	NWGC	3R01HL098433-05S1	WGS
**phs001368**	CHS	CHS	3	Baylor	HHSN268201600033I	WGS
**phs001368**	CHS	CHS	5.5-5.6; 6 backfill	Broad Genomics	HHSN268201600034I	WGS
**phs001368**	VTE	CHS VTE	2	Baylor	3U54HG003273-12S2 / HHSN268201500015C	WGS
**phs001412**	AA_CAC	DHS	2	Broad Genomics	HHSN268201500014C	WGS

**phs000974**	AFGen	FHS AFGen	1	Broad Genomics	3R01HL092577-06S1	WGS
**phs000974**	FHS	FHS	pilot; 4.5; 5.5-5.6; 6 backfill	Broad Genomics	HHSN268201600034I	WGS
**phs000974**	FHS	FHS	1	Broad Genomics	3U54HG003067-12S2	WGS
**phs001218**	AA_CAC	GeneSTAR AA_CAC	2	Broad Genomics	HHSN268201500014C	WGS
**phs001218**	GeneSTAR	GeneSTAR	2	Psomagen	3R01HL112064-04S1	WGS
**phs001218**	GeneSTAR	GeneSTAR	legacy	Illumina	R01HL112064	WGS
**phs001345**	HyperGEN_GENOA	GENOA	2	NWGC	3R01HL055673-18S1	WGS
**phs001345**	AA_CAC	GENOA AA_CAC	2	Broad Genomics	HHSN268201500014C	WGS
**phs001217**	GenSalt	GenSalt	2	Baylor	HHSN268201500015C	WGS
**phs001359**	GOLDN	GOLDN	2	NWGC	3R01HL104135-04S1	WGS
**phs001395**	HCHS_SOL	HCHS_SOL	3	Baylor	HHSN268201600033I	WGS
**phs001293**	HyperGEN_GENOA	HyperGEN	2	NWGC	3R01HL055673-18S1	WGS
**phs000964**	JHS	JHS	1	NWGC	HHSN268201100037C	WGS
**phs001416**	AA_CAC	MESA AA_CAC	2	Broad Genomics	HHSN268201500014C	WGS
**phs001416**	MESA	MESA	2	Broad Genomics	3U54HG003067-13S1	WGS

**phs001062**	AFGen	MGH_AF	1	Broad Genomics	3R01HL092577-06S1	WGS
**phs001062**	AFGen	MGH_AF	1.4; 1.5; 2.4	Broad Genomics	3U54HG003067-12S2 / 3U54HG003067-13S1; 3U54HG003067-12S2 / 3U54HG003067-13S1; 3UM1HG008895-01S2	WGS
**phs001215**	SAFS	SAFS	legacy	Illumina	R01HL113322	WGS
**phs001215**	SAFS	SAFS	1	Illumina	3R01HL113323-03S1	WGS
**phs000972**	Samoan	Samoan	2	NYGC Genomics	HHSN268201500016C	WGS
**phs000972**	Samoan	Samoan	1	NWGC	HHSN268201100037C	WGS
**phs001387**	THRV	THRV	2	Baylor	3R01HL111249-04S1 / HHSN26820150015C	WGS
**phs001237**	WHI	WHI	2	Broad Genomics	HHSN268201500014C	WGS

- Baylor = Baylor College of Medicine Human Genome Sequencing Center
- Broad Genomics = Broad Institute Genomics Platform
- Broad Metabolomics = Broad Institute and Beth Israel Metabolomics Platform
- Illumina = Illumina
- Keck MGC = Keck Molecular Genomics Core Facility
- MGI = McDonnell Genome Institute
- NWGC = Northwest Genomics Center
- NYGC Genomics = New York Genome Center Genomics
- Psomagen = Psomagen

Supplementary Figures:

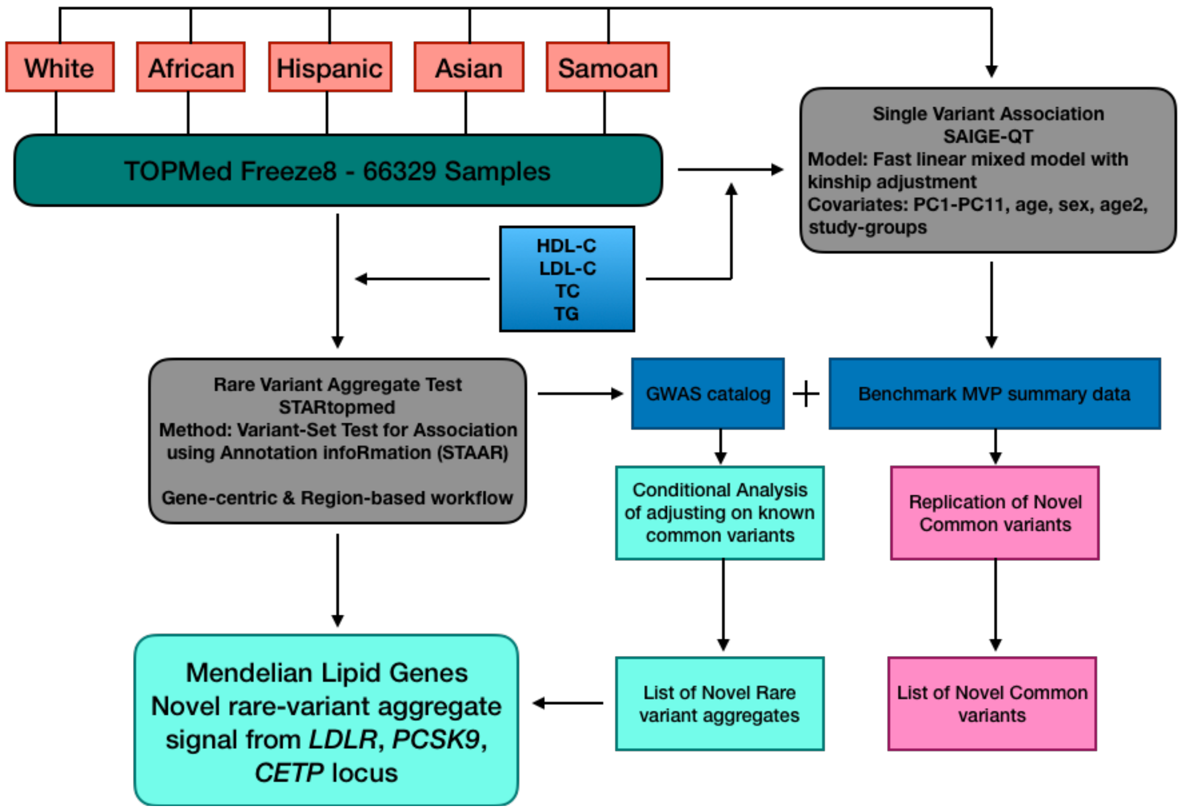

Supplementary Fig. 1:

Overview of the workflow implement in the study. Multi-ancestral whole genome sequenced data from TOPMed freeze 8, including 66329 samples were qualified for the analysis from five different ancestral groups, were analyzed with lipids. Single variant GWAS and genome-wide rare variant aggregates tests were conducted using SAIGE and STAAR methods, respectively. The results were compared with the GWAS catalog and MVP summary stats to identify novel associations. Conditional analyses of known associated single variants were conducted for rare variant association tests at known loci. Mendelian lipid loci were explored in further detail.

TOPMed – Trans-Omics for Precision Medicine; GWAS – Genome Wide Association Study; SAIGE – Scalable and Accurate Implementation of GEneralized mixed model; STAAR – variant-Set Test for Association using Annotation information; MVP – Million Veteran Program

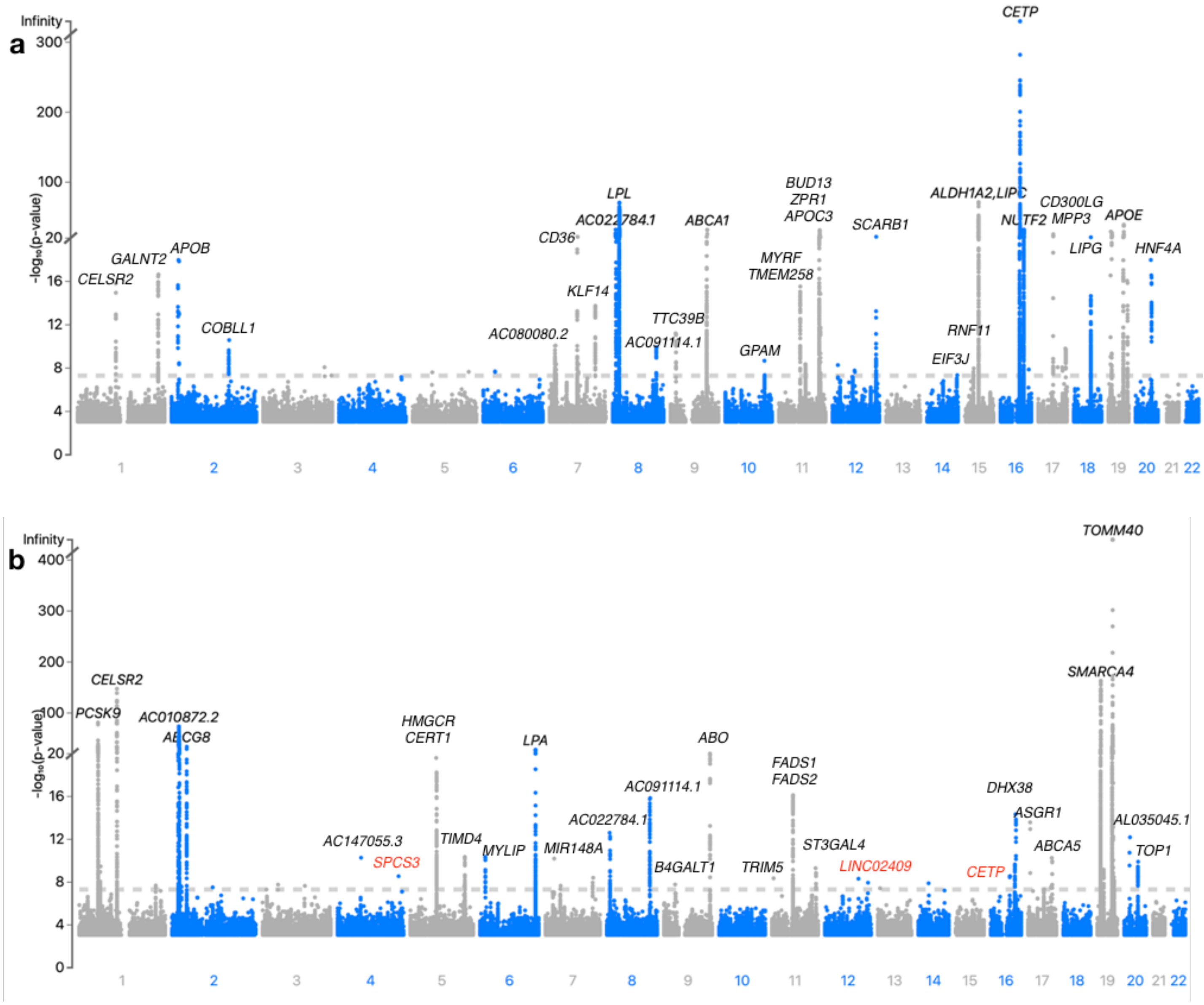
a
b
-log₁₀(p-value)
Infinity
CETP
LPL
AC022784.1
ABCA1
BUD13
ZPR1
APOC3
SCARB1
ALDH1A2,LIPC
NUTF2
CD300LG
MPP3
APOE
CD36
GALNT2
APOB
CELSR2
KLF14
MYRF
TMEM258
LIPG
HNF4A
COBLL1
AC080080.2
TTC39B
AC091114.1
GPAM
RNF11
EIF3J
TOMM40
SMARCA4
PCSK9
AC010872.2
ABCG8
HMGCR
CERT1
LPA
ABO
FADS1
FADS2
DHX38
ASGR1
AL035045.1
AC147055.3
SPCS3
TIMD4
MYLIP
MIR148A
AC022784.1
B4GALT1
TRIM5
ST3GAL4
LINC02409
CETP
ABCA5
TOP1

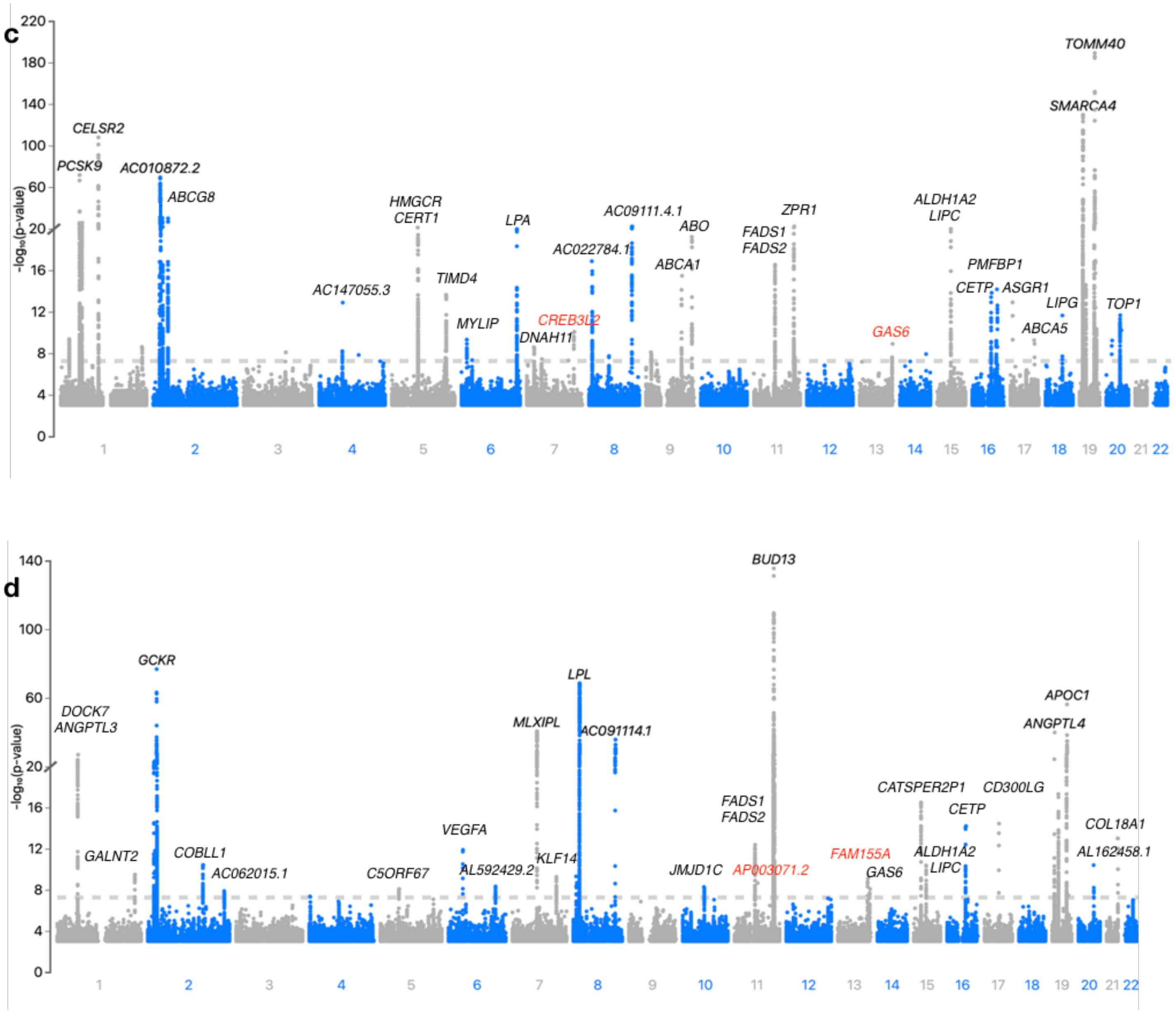

Supplementary Fig. 2:

Manhattan plots for the four lipid phenotypes. MH plots for single variant GWAS for the four lipids. Genes nearby to the variants that are strongly associated at a locus are mapped on the plot for each chromosome. Genes linked to SNPs at potentially novel loci in TOPMed are noted in red. **a**, MH plots for HDL-C association. **b**, MH plots for LDL-C association. **c**, MH plots for TC association. **d**, MH plots for TG association. MH – Manhattan; GWAS – Genome Wide Association Study; SNPs – Single Nucleotide Polymorphisms; TOPMed – Trans-Omics for Precision Medicine; HDL-C – High-Density Lipoprotein Cholesterol; LDL-C – Low-Density Lipoprotein Cholesterol; TC – Total Cholesterol; TG – Triglycerides.

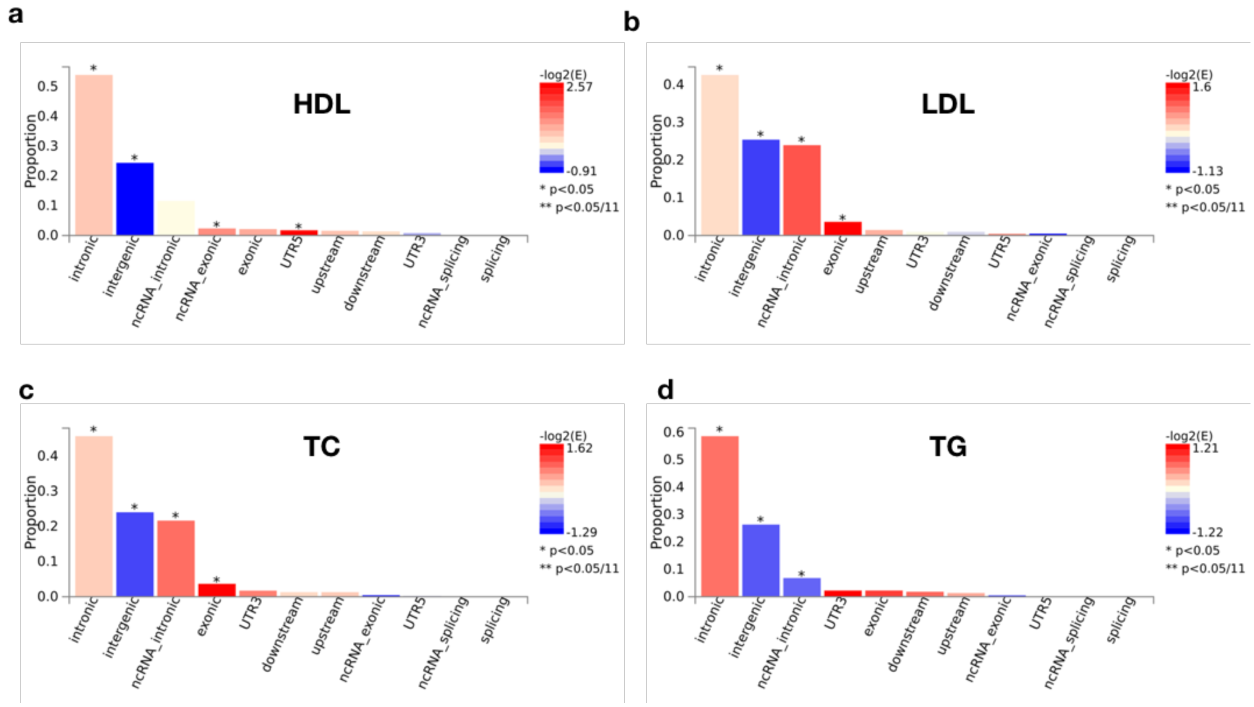

Supplementary Fig. 3

Distribution of variants into different genomic regions. Enrichment analysis was carried out for all the variants present in the significant genomic loci. Summary statistics from two-sided genetic association testing preformed using SAIGE-QT model from all biologically independent samples (N=66,329) were used for this analysis. Most of the variants were present in the non-coding region of the genome for all lipid enrichment. Variants were enriched in the intergenic region of the genome **a,** Distribution of significant variants from HDL association (intergenic region: p-value=$9.3x10^{-24}$). **b,** Distribution of significant variants from LDL association (intergenic region: p-value=$6.9x10^{-18}$). **c,** Distribution of significant variants from TC association (intergenic region: p-value=$1.4x10^{-22}$). **d,** Distribution of significant variants from TG association (intergenic region: p-value=$2.5x10^{-09}$).

HDL-C – High-Density Lipoprotein Cholesterol; LDL-C – Low-Density Lipoprotein Cholesterol; TC – Total Cholesterol; TG – Triglycerides; UTR – untranslated region.

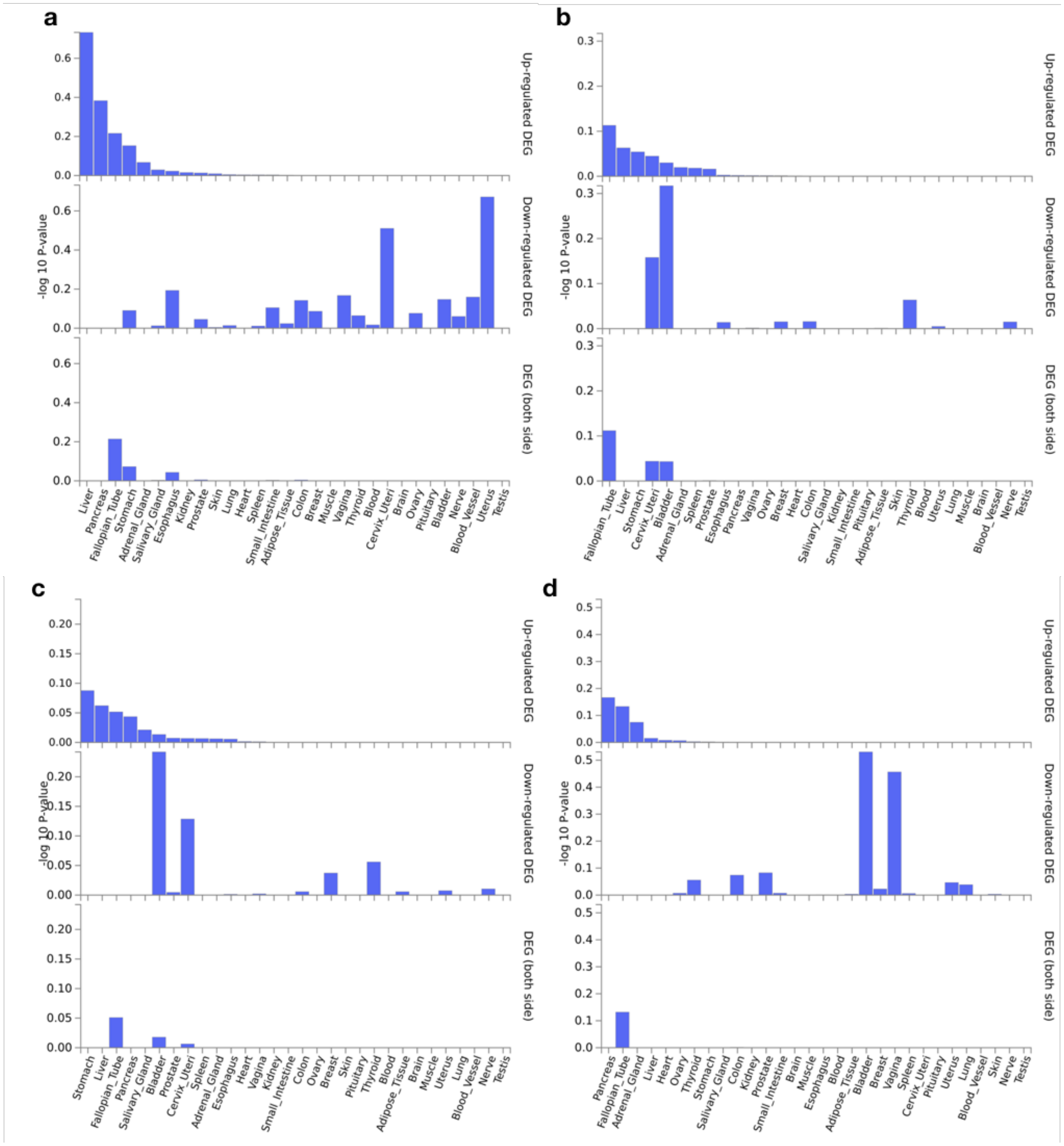

Supplementary Fig. 4

eQTL enrichment using GTEx datasets. Enrichment of eQTLs were carried out using multiple tissue data from GTEx database using GENE2FUNC function in FUMA workflow. eQTLs were enriched in few important tissue types relevant to lipid phenotypes. **a,** Distribution of eQTLs from HDL association. **b,** Distribution of eQTLs from LDL association. **c,** Distribution of eQTLs from TC association. **d,** Distribution of eQTLs from TG association.

eQTL – Expression quantitative Trait Loci; GTEx – Genotype-Tissue Expression; HDL-C – High-Density Lipoprotein Cholesterol; LDL-C – Low-Density Lipoprotein Cholesterol; TC – Total Cholesterol; TG – Triglycerides.

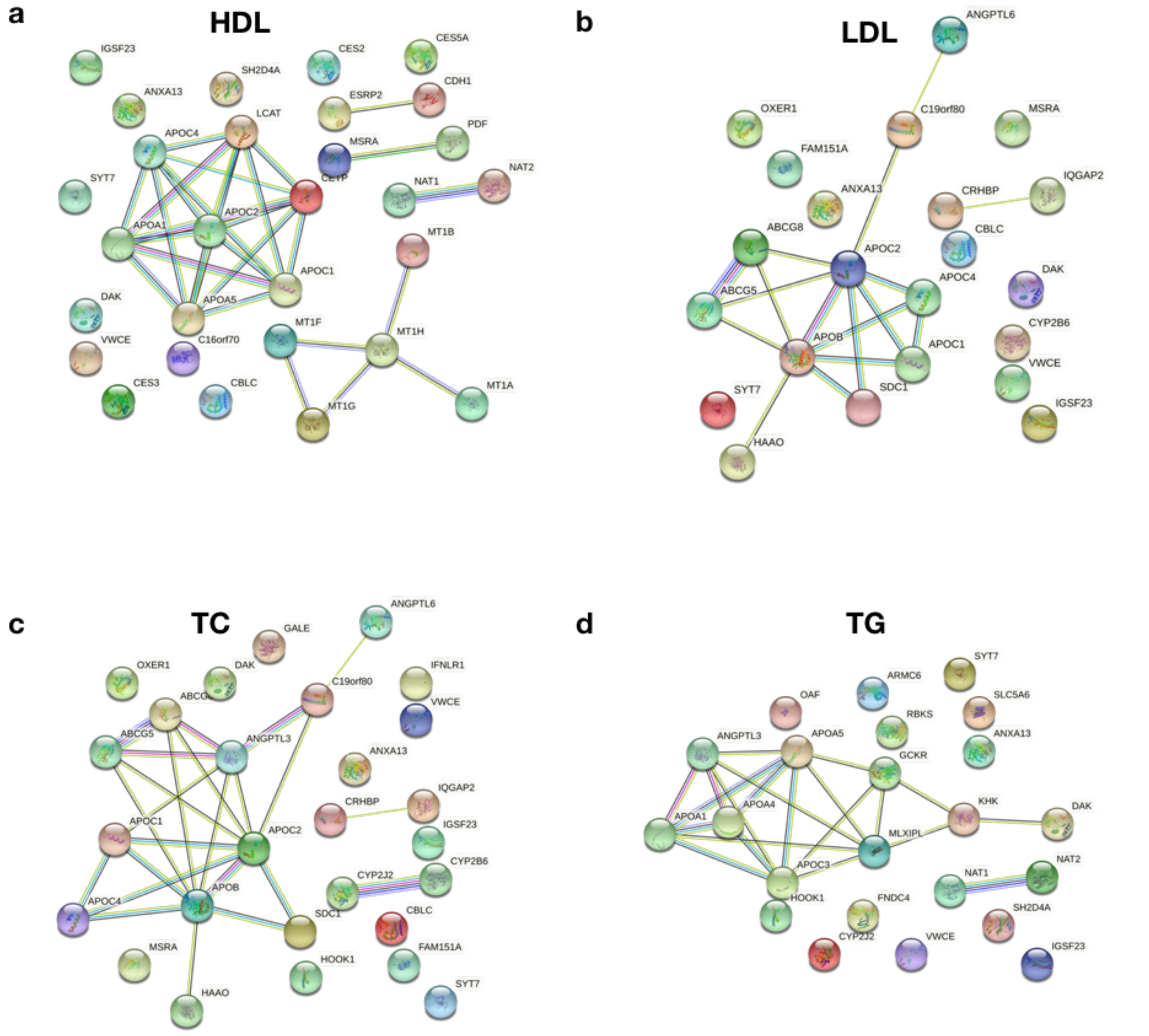

Supplementary Fig. 5
Protein-protein interaction network of the enriched genes from liver tissue. Genes identified to be significantly enriched in Liver tissue from GTEx analysis were mapped to the protein-protein interaction network from STRING database.
a, Network of genes from HDL association. **b,** Network of genes from LDL association. **c,** Network of genes from TC association. **d,** Network of genes from TG association. GTEx – Genotype-Tissue Expression; HDL-C – High-Density Lipoprotein Cholesterol; LDL-C – Low-Density Lipoprotein Cholesterol; TC – Total Cholesterol; TG – Triglycerides.

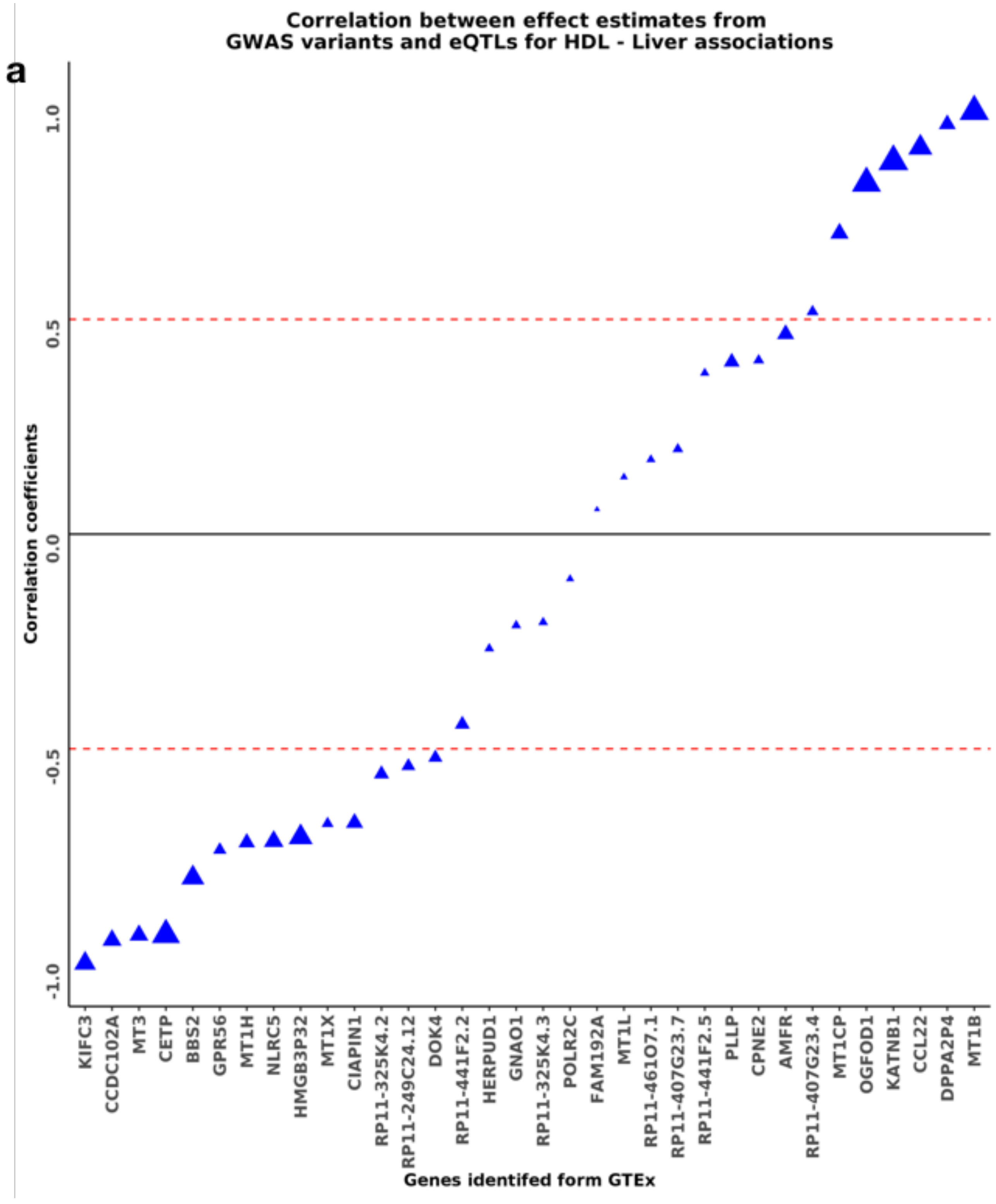

Correlation between effect estimates from
GWAS variants and eQTLs for HDL - Liver associations
a
Correlation coefficients
1.0
0.5
0.0
-0.5
-1.0
KIFC3
CCDC102A
MT3
CETP
BBS2
GPR56
MT1H
NLRC5
HMGB3P32
MT1X
CIAPIN1
RP11-325K4.2
RP11-249C24.12
DOK4
RP11-441F2.2
HERPUD1
GNAO1
RP11-325K4.3
POLR2C
FAM192A
MT1L
RP11-461O7.1
RP11-407G23.7
RP11-441F2.5
PLLP
CPNE2
AMFR
RP11-407G23.4
MT1CP
OGFOD1
KATNB1
CCL22
DPPA2P4
MT1B
Genes identifed form GTEx

b

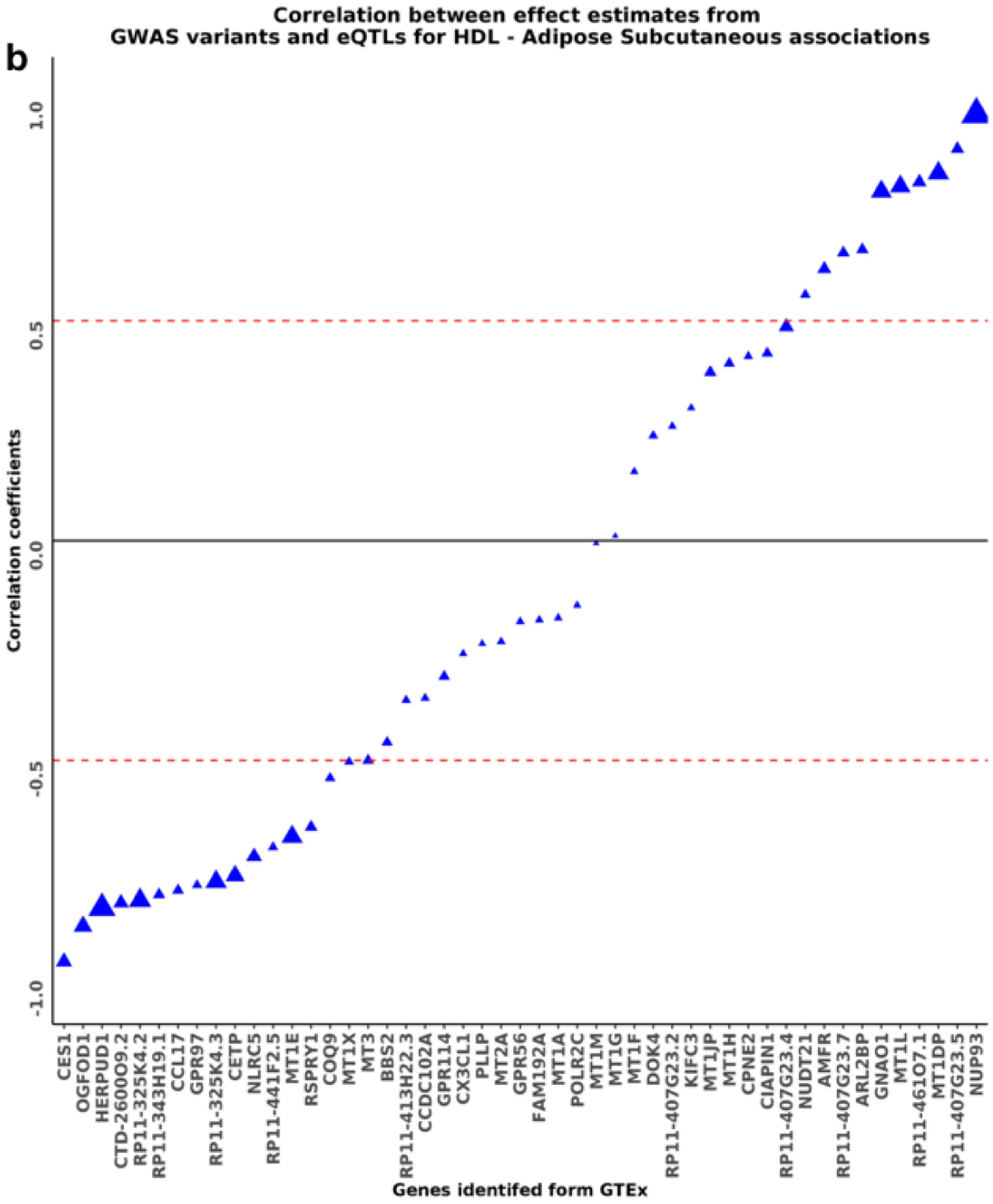
Correlation between effect estimates from
GWAS variants and eQTLs for HDL - Adipose Subcutaneous associations
Correlation coefficients
1.0
0.5
0.0
-0.5
-1.0
CES1
OGFOD1
HERPUD1
CTD-2600O9.2
RP11-325K4.2
RP11-343H19.1
CCL17
GPR97
RP11-325K4.3
CETP
NLRC5
RP11-441F2.5
MT1E
RSPRY1
COQ9
MT1X
MT3
BBS2
RP11-413H22.3
CCDC102A
GPR114
CX3CL1
PLLP
MT2A
GPR56
FAM192A
MT1A
POLR2C
MT1M
MT1G
MT1F
DOK4
RP11-407G23.2
KIFC3
MT1JP
MT1H
CPNE2
CIAPIN1
RP11-407G23.4
NUDT21
AMFR
RP11-407G23.7
ARL2BP
GNAO1
MT1L
RP11-461O7.1
MT1DP
RP11-407G23.5
NUP93
Genes identifed form GTEx

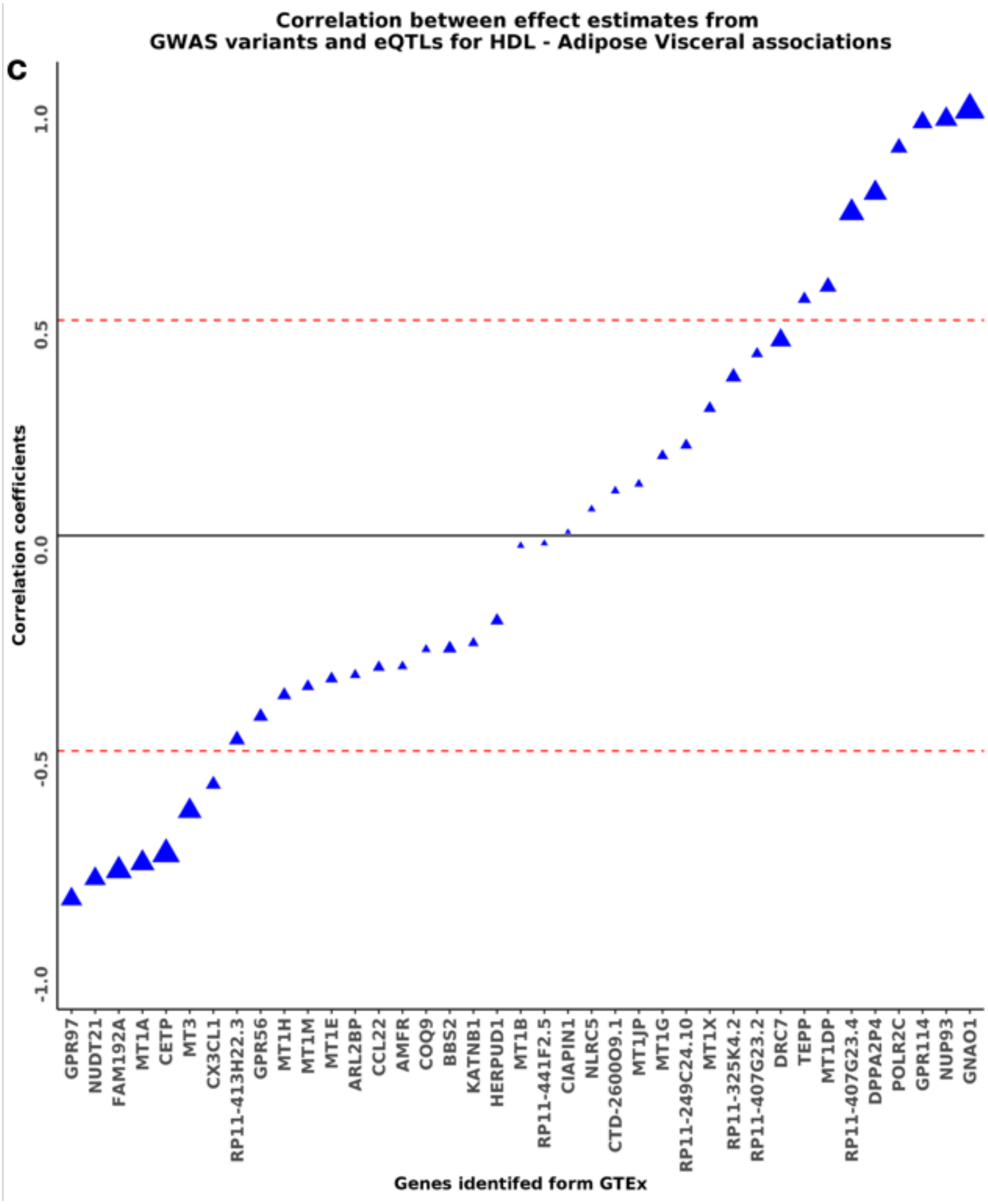

Supplementary Fig. 6:
Correlation of *CETP* gene expression effects with HDL-C effects. The effects from GWAS and GTEx data from Liver, Adipose Subcutaneous and Adipose Visceral tissues

for the eQTLs were correlated. GWAS from all biologically independent samples (N=66,329) were included for this analysis. Two-sided Pearson correlation test was implemented. The x-axis contains each gene linked to the eQTLs and y-axis represents the correlation coefficients, where the x-axis is ordered based on the correlation coefficients and size of each datapoint is based on the p-value of the correlation test. *CETP* along with other genes have a strong negative correlation of the effect estimates. **A**, Liver tissue data **b**, Adipose Subcutaneous tissue data **c**, Adipose Visceral (Omentum) tissue data.

GWAS – Genome Wide Association Study; GTEx – Genotype-Tissue Expression; eQTLs – Expression Quantitative Trait Loci; HDL-C – High-Density Lipoprotein Cholesterol.

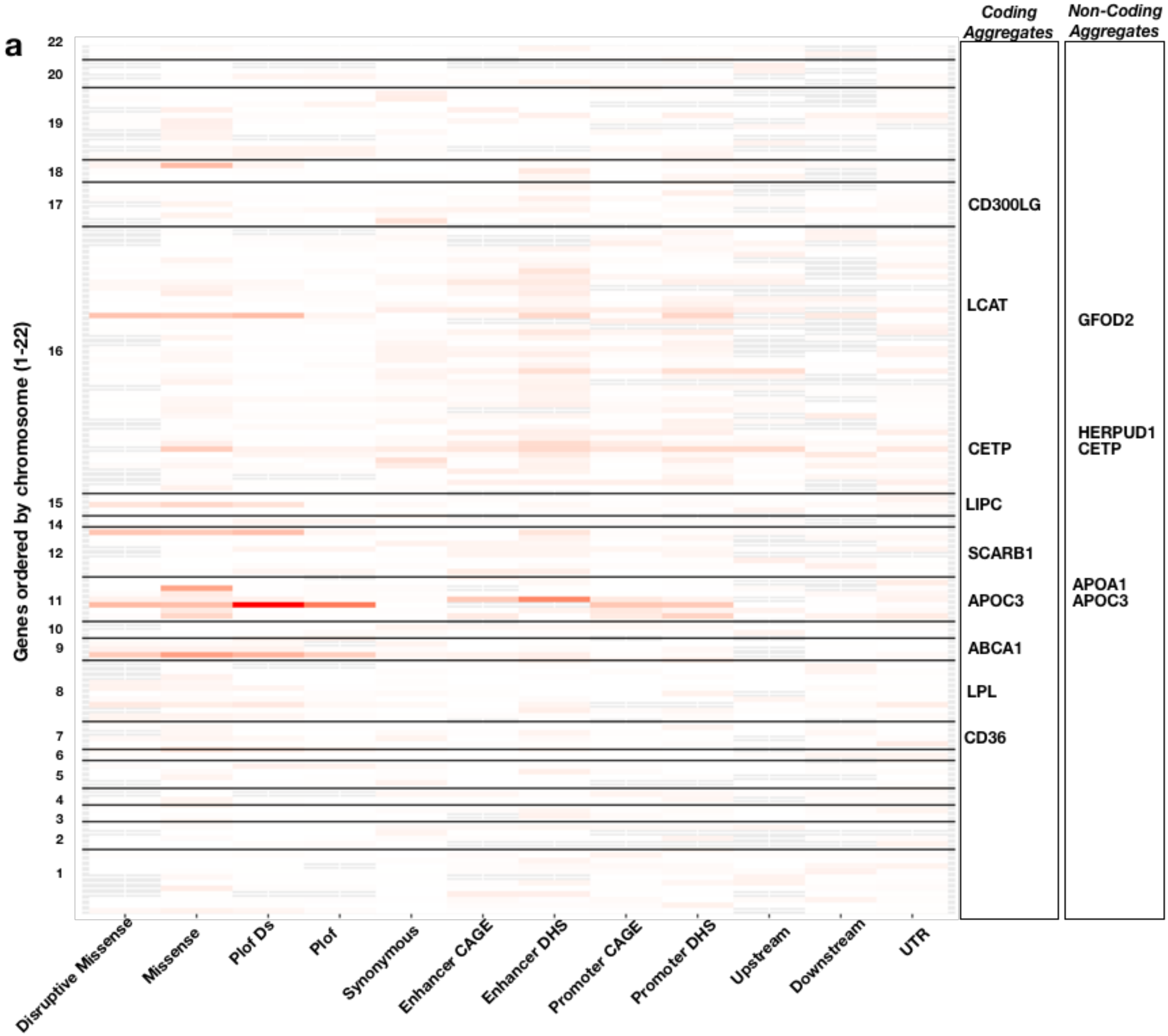
a
Coding Aggregates
Non-Coding Aggregates
Genes ordered by chromosome (1-22)
22
20
19
18
17
16
15
14
12
11
10
9
8
7
6
5
4
3
2
1
CD300LG
LCAT
CETP
LIPC
SCARB1
APOC3
ABCA1
LPL
CD36
GFOD2
HERPUD1
CETP
APOA1
APOC3
Disruptive Missense
Missense
Plof Ds
Plof
Synonymous
Enhancer CAGE
Enhancer DHS
Promoter CAGE
Promoter DHS
Upstream
Downstream
UTR

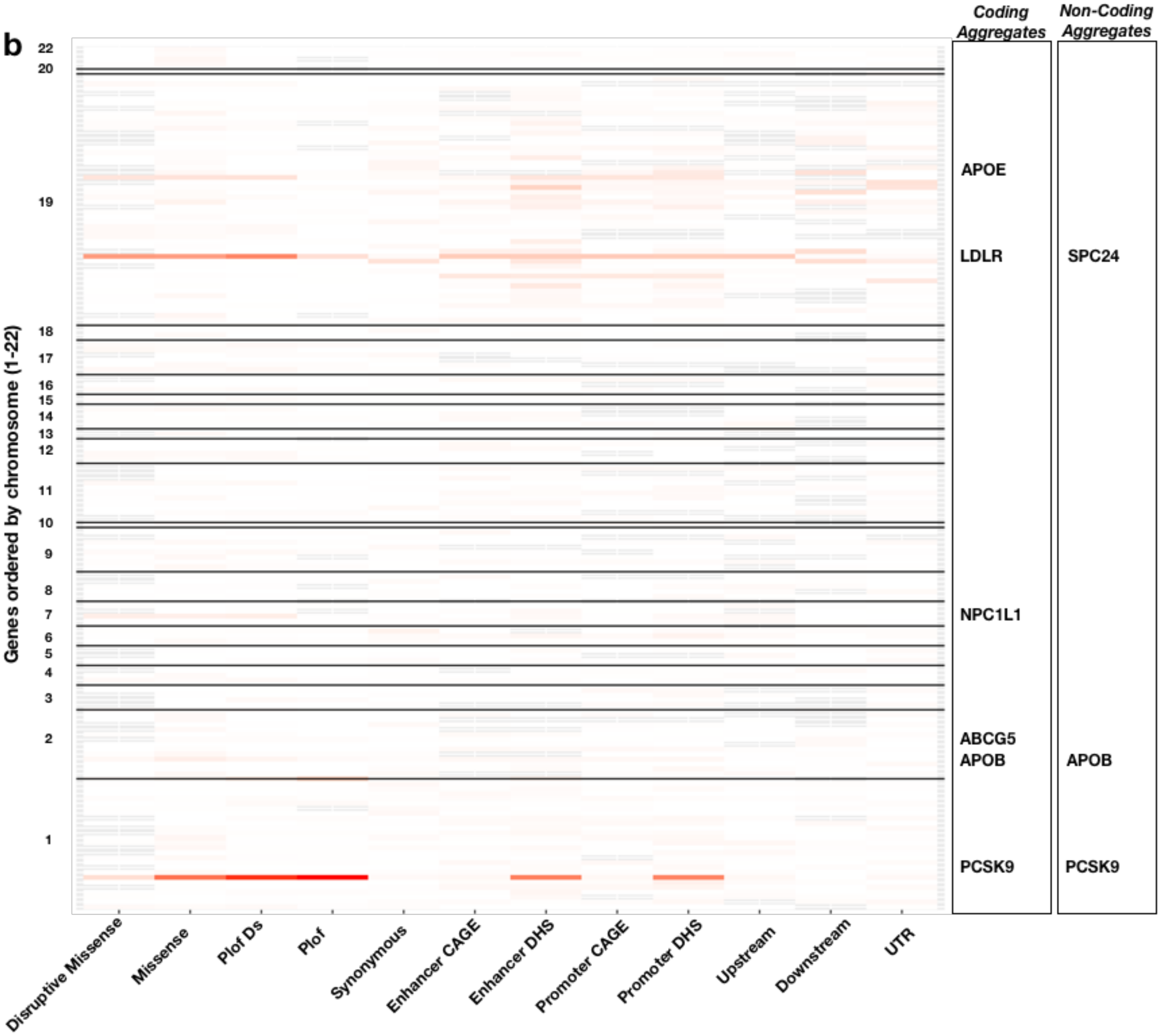
b
Coding Aggregates
Non-Coding Aggregates
Genes ordered by chromosome (1-22)
22
20
19
18
17
16
15
14
13
12
11
10
9
8
7
6
5
4
3
2
1
APOE
LDLR
SPC24
NPC1L1
ABCG5
APOB
APOB
PCSK9
PCSK9
Disruptive Missense
Missense
Plof Ds
Plof
Synonymous
Enhancer CAGE
Enhancer DHS
Promoter CAGE
Promoter DHS
Upstream
Downstream
UTR

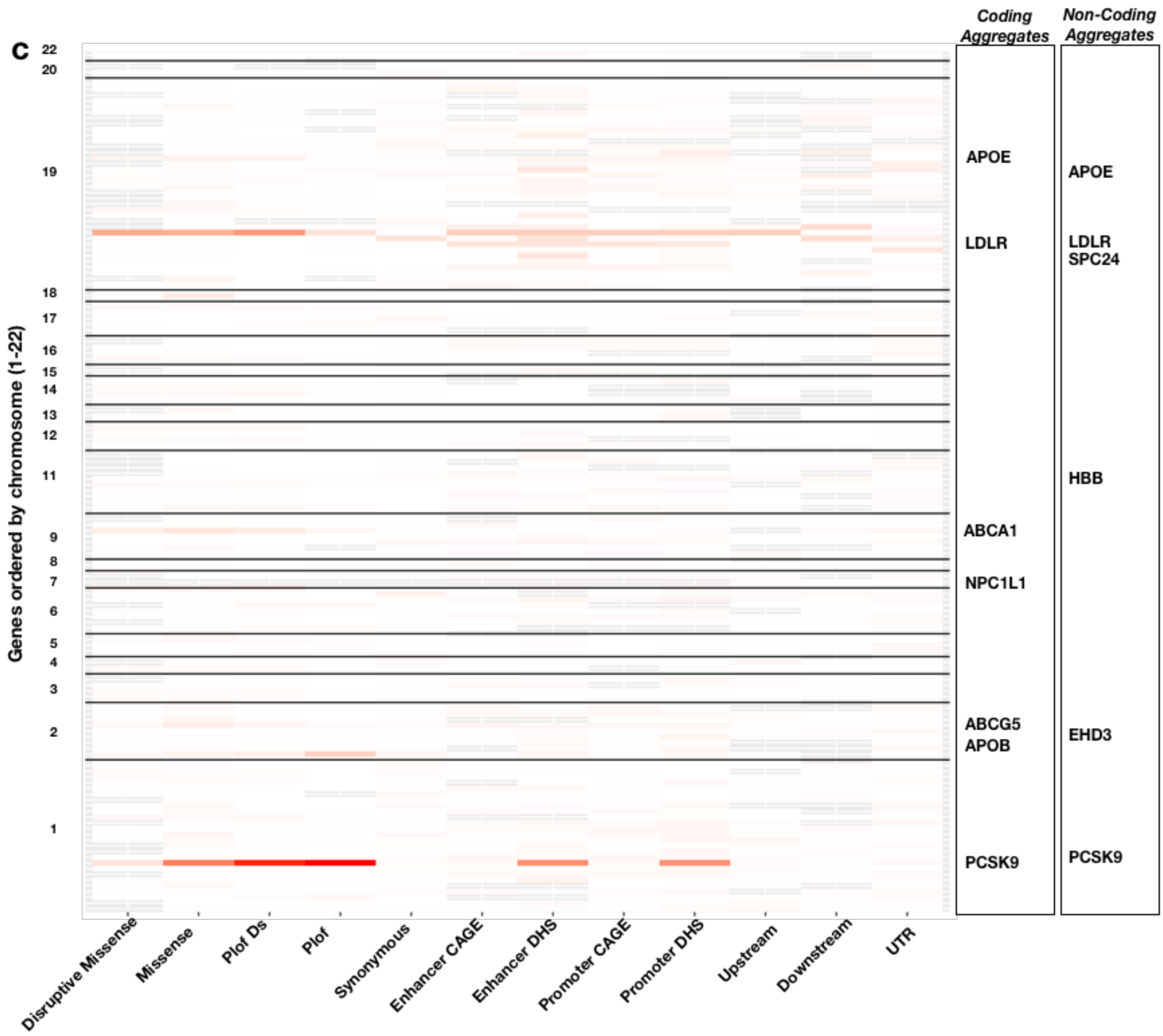
C
Genes ordered by chromosome (1-22)
22
20
19
18
17
16
15
14
13
12
11
9
8
7
6
5
4
3
2
1
Disruptive Missense
Missense
Plof Ds
Plof
Synonymous
Enhancer CAGE
Enhancer DHS
Promoter CAGE
Promoter DHS
Upstream
Downstream
UTR
Coding Aggregates
APOE
LDLR
ABCA1
NPC1L1
ABCG5
APOB
PCSK9
Non-Coding Aggregates
APOE
LDLR
SPC24
HBB
EHD3
PCSK9

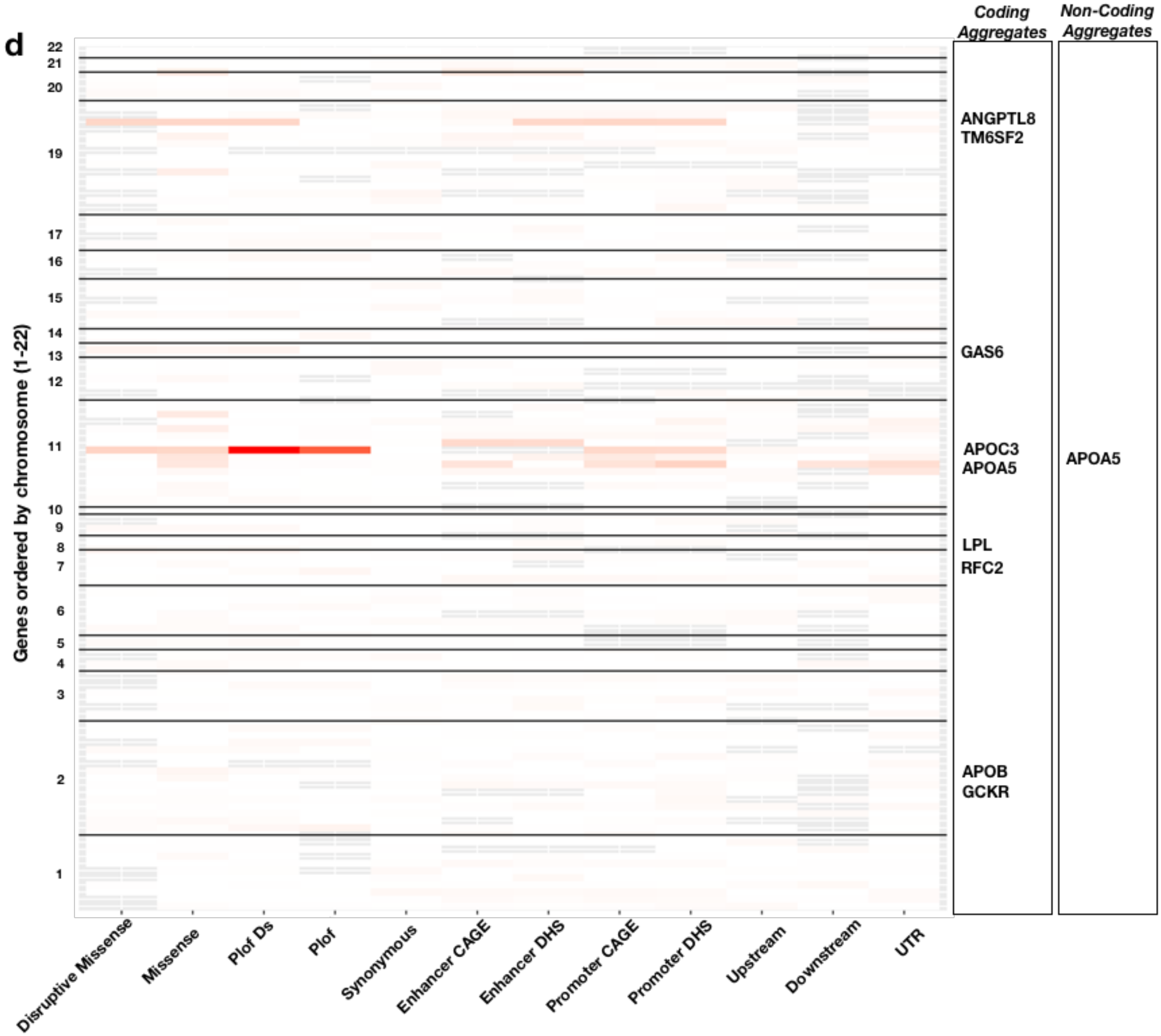

Supplementary Fig. 7

Genome wide view of coding and non-coding aggregate sets. Aggregate sets that were significant with STAAR-O p-values lesser than 5E-04 were retained. The genes are ordered based on chromosomes, following by their genomic positions. The coloring scale is based on -log10(STAAR-O p-values). The most significant mask has bright red tile. Aggerate sets significant after conditional analysis is represented with the corresponding genes on the right panel, for coding and non-coding aggregates. The p-

values were calculated from two-sided aggregate testing preformed using STAAR gene-centric model, where the model was adjusted for all the covariates. **A**, Rare variant aggregates for HDL-C. **b**, Rare variant aggregates for LDL-C. **c**, Rare variant aggregates for TC. **D**, Rare variant aggregates for TG.
STAAR – variant-Set Test for Association using Annotation information; HDL-C – High-Density Lipoprotein Cholesterol; LDL-C – Low-Density Lipoprotein Cholesterol; TC – Total Cholesterol; TG – Triglycerides.

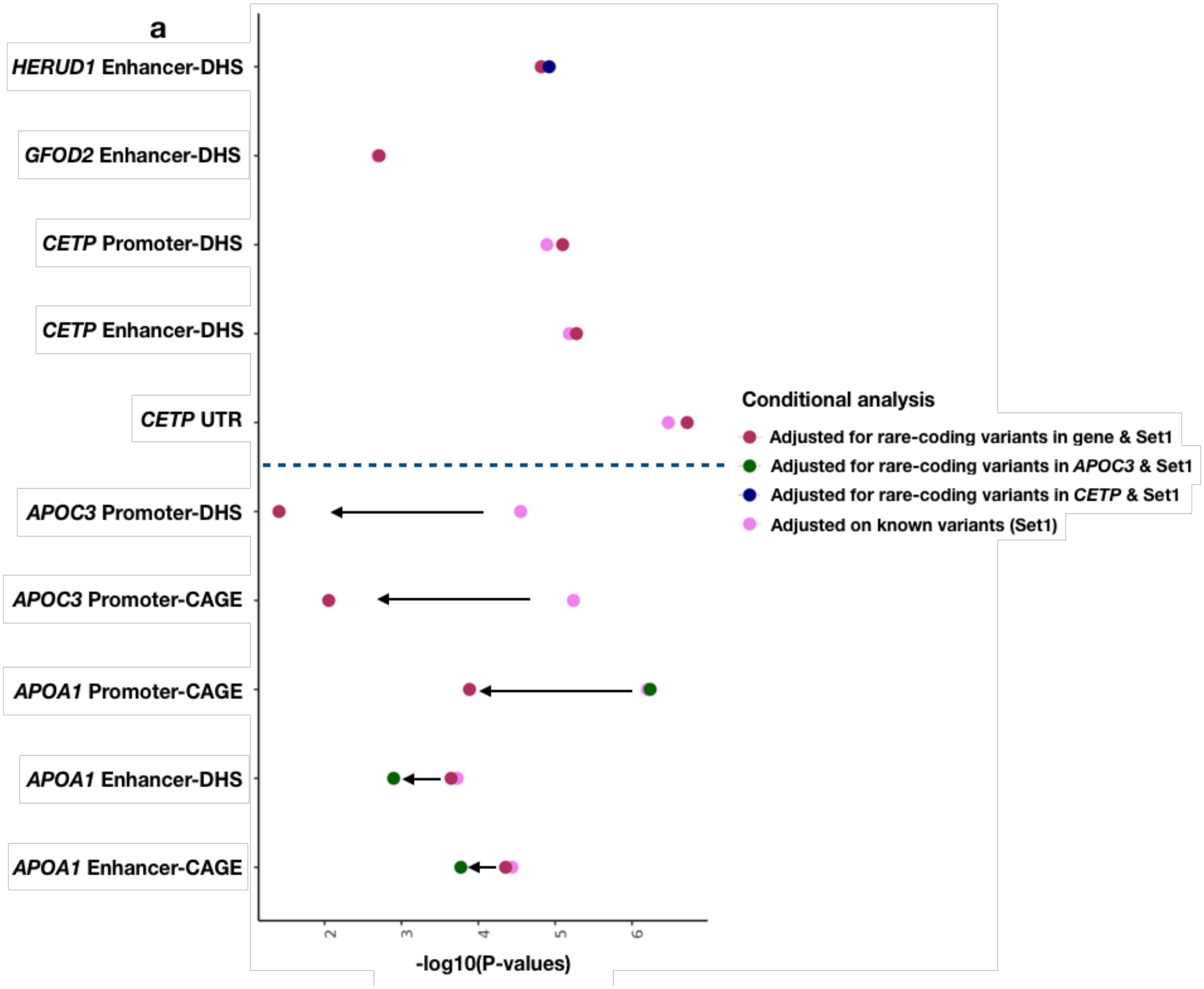

a
HERUD1 Enhancer-DHS
GFOD2 Enhancer-DHS
CETP Promoter-DHS
CETP Enhancer-DHS
CETP UTR
APOC3 Promoter-DHS
APOC3 Promoter-CAGE
APOA1 Promoter-CAGE
APOA1 Enhancer-DHS
APOA1 Enhancer-CAGE
2
3
4
5
6
-log10(P-values)
Conditional analysis
Adjusted for rare-coding variants in gene & Set1
Adjusted for rare-coding variants in APOC3 & Set1
Adjusted for rare-coding variants in CETP & Set1
Adjusted on known variants (Set1)

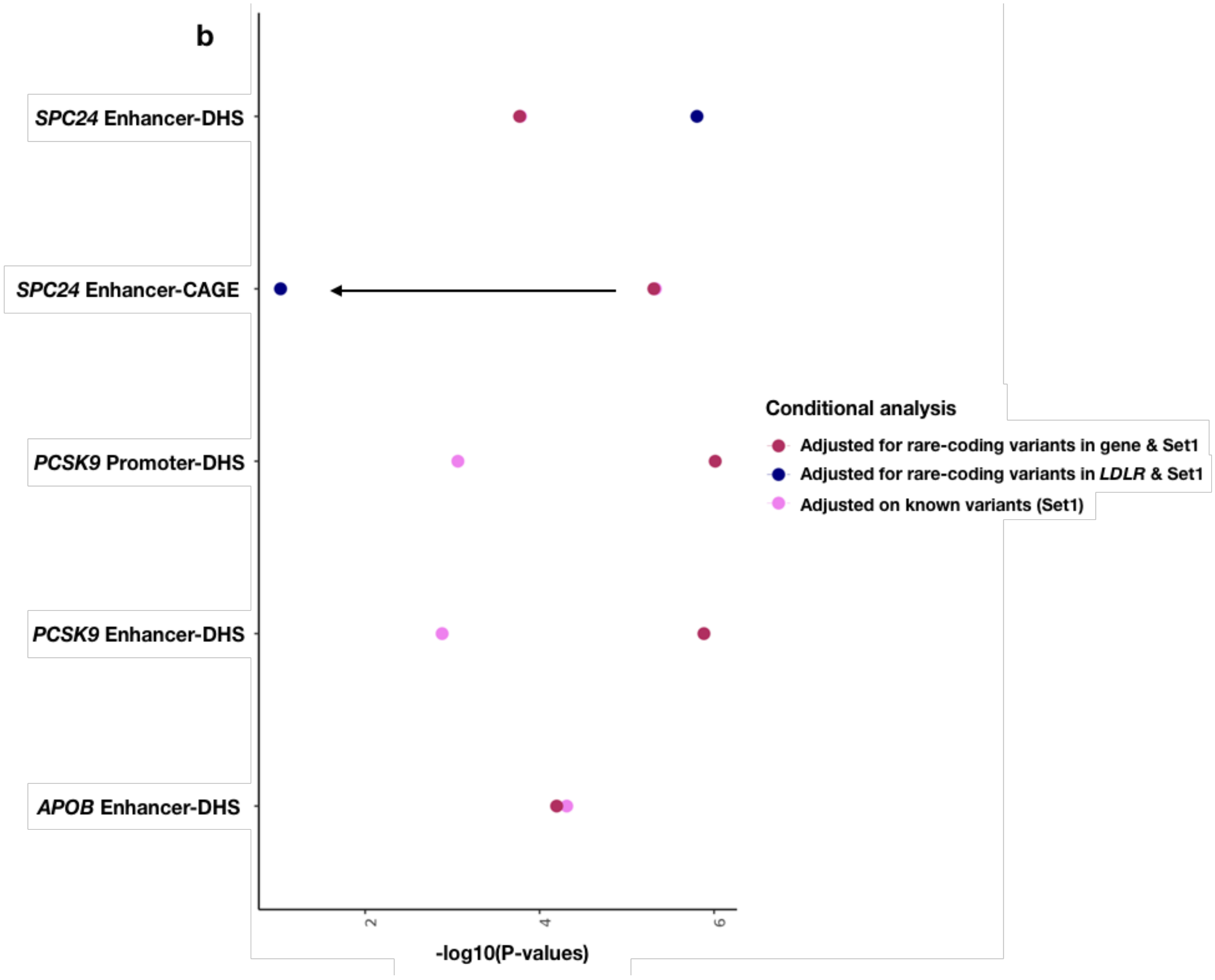

Supplementary Fig. 8
Conditional analysis of coding rare-variants from the same gene and a near-by gene. Non-coding rare variant sets significantly associated with HDL-C and LDL-C after the conditional analysis on known variants are shown with additional adjustment on rare-coding variants. The different colored dots on the plot represents the conditional STAAR-O p-values when adjusting for known variants (Set1) and rare-coding variants of the same or near-by gene. All biologically independent samples (N=66,329) were included in this analysis. The p-values were calculated from two-sided aggregate testing preformed using STAAR gene-centric model, where the model was adjusted for all the covariates**. a**, Non-coding rare variant sets significantly associated with HDL-C. Conditional analysis shows that the signal from *APOC3* and *APOA1* drop after it was adjusted for coding rare-variants. Whereas the signal from *CETP* locus combined with *HERPUD1* variant sets are retained. Similarly, *GFOD2* non-coding rare-variant aggregate is significant, where there is no change in STAAR-O p-values after the

conditional analysis. The dotted line separates the variants sets that are significant and insignificant after the additional conditional analysis. **b**, Non-coding rare variant sets significantly associated with LDL-C. After adjustment, *APOB* signal drops minimally, whereas *PCSK9* signal enhances. Conditioning on the near-by gene *LDLR*, the signal from *SPC24* enhancer-DHS improves, but enhancer-CAGE drops significantly.
STAAR – variant-Set Test for Association using Annotation information; HDL-C – High-Density Lipoprotein Cholesterol; LDL-C – Low-Density Lipoprotein Cholesterol; CAGE – Cap Analysis of Gene Expression; DHS – DNase hypersensitivity.

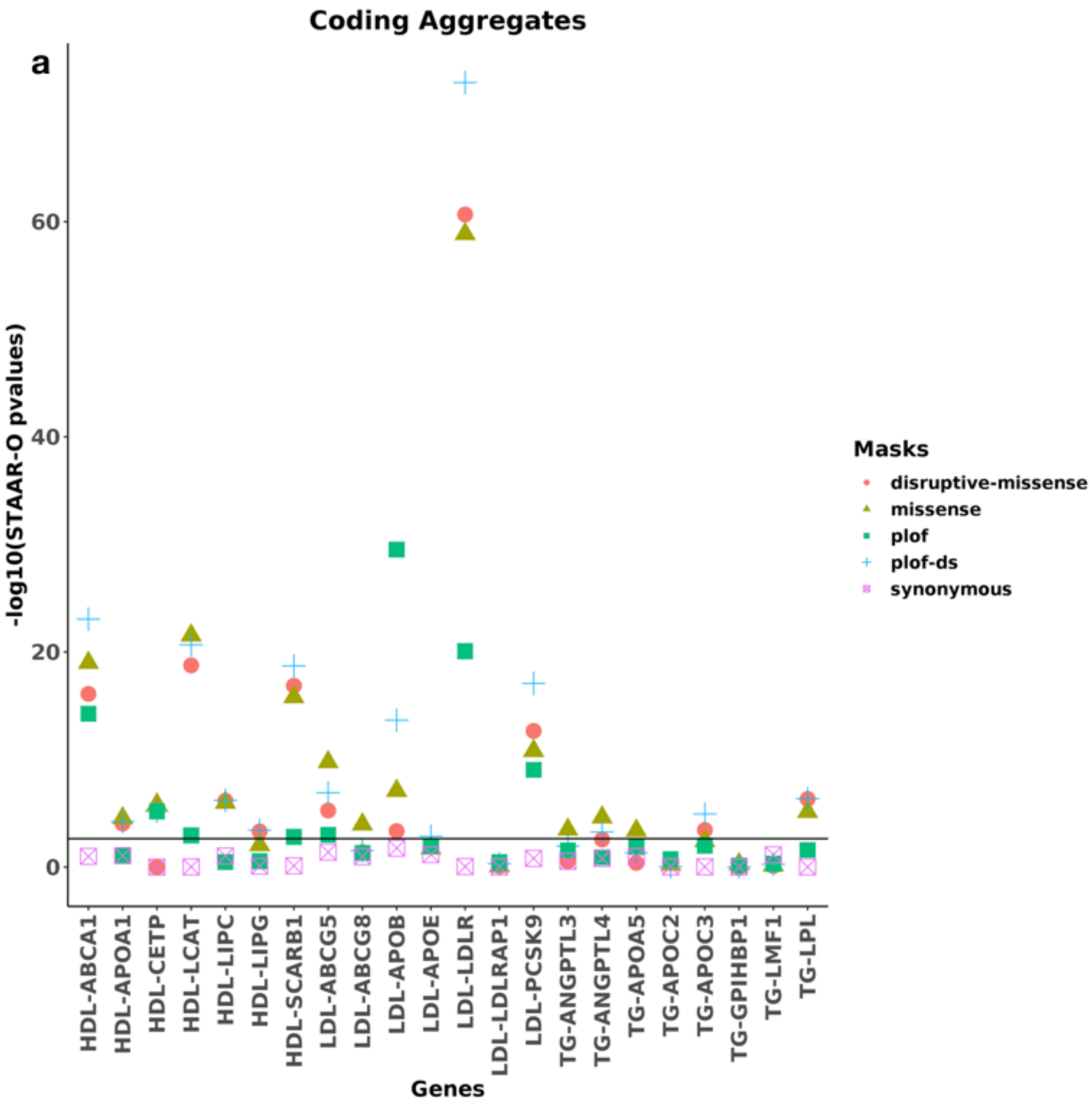
Coding Aggregates
a
-log10(STAAR-O pvalues)
60
40
20
0
Masks
disruptive-missense
missense
plof
plof-ds
synonymous
HDL-ABCA1
HDL-APOA1
HDL-CETP
HDL-LCAT
HDL-LIPC
HDL-LIPG
HDL-SCARB1
LDL-ABCG5
LDL-ABCG8
LDL-APOB
LDL-APOE
LDL-LDLR
LDL-LDLRAP1
LDL-PCSK9
TG-ANGPTL3
TG-ANGPTL4
TG-APOA5
TG-APOC2
TG-APOC3
TG-GPIHBP1
TG-LMF1
TG-LPL
Genes

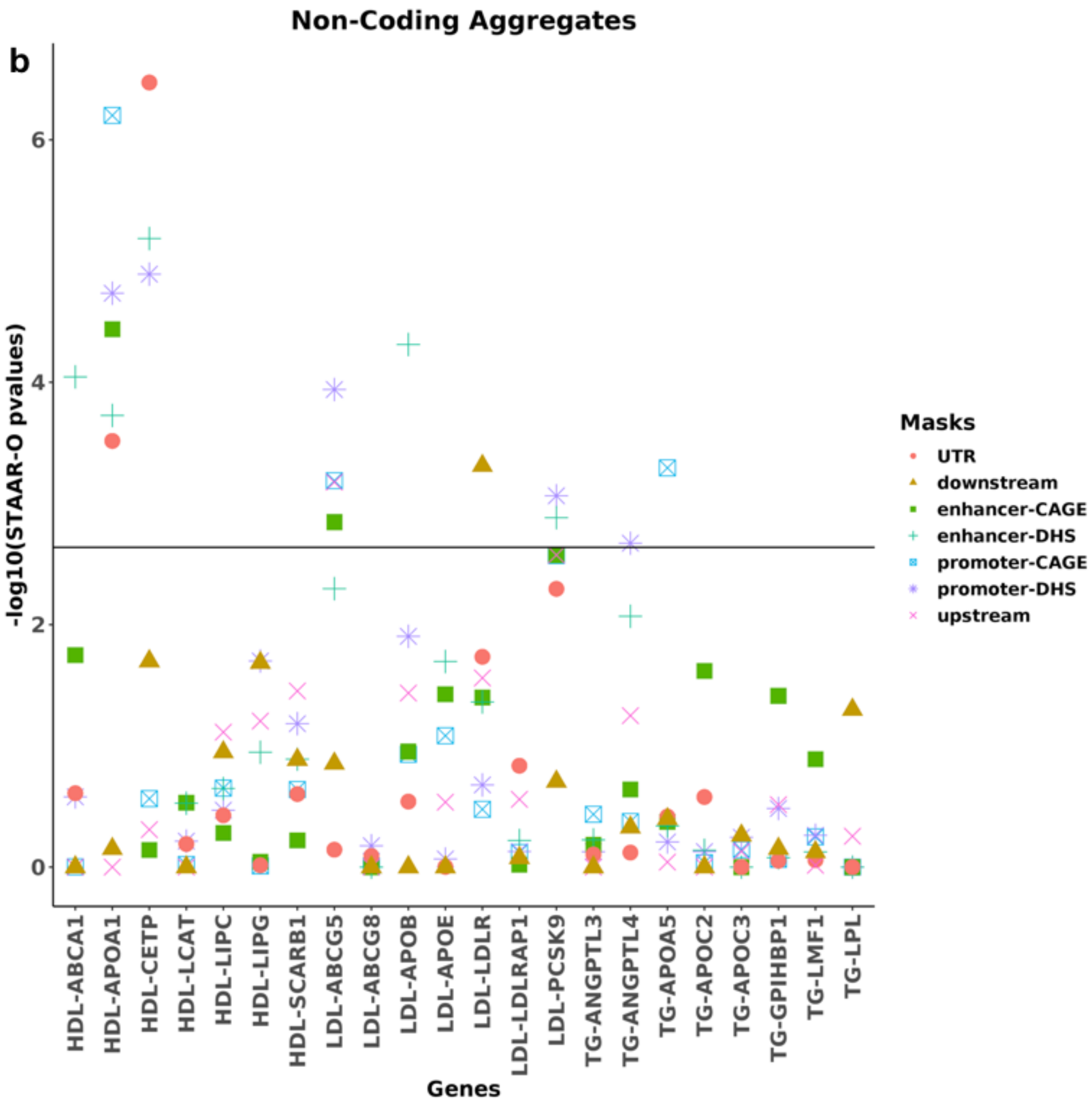

Supplementary Fig. 9

Distribution of Mendelian gene STAAR-O p-values. The 22 genes are ordered based on the lipid categories (HDL-C, LDL-C and TG) and the most significant association of each gene aggregate set is represented as p-values. All biologically independent samples (N=66,329) were included in this analysis. The p-values were calculated from two-sided aggregate testing preformed using STAAR gene-centric model, where the model was adjusted for all the covariates. **a**, -log10(STAAR-O p-values) of the five

different coding masks. **b**, -log10(STAAR-O p-values) of the seven different non-coding masks.
STAAR – variant-Set Test for Association using Annotation information; LDL-C – Low-Density Lipoprotein Cholesterol; TG – Triglycerides.

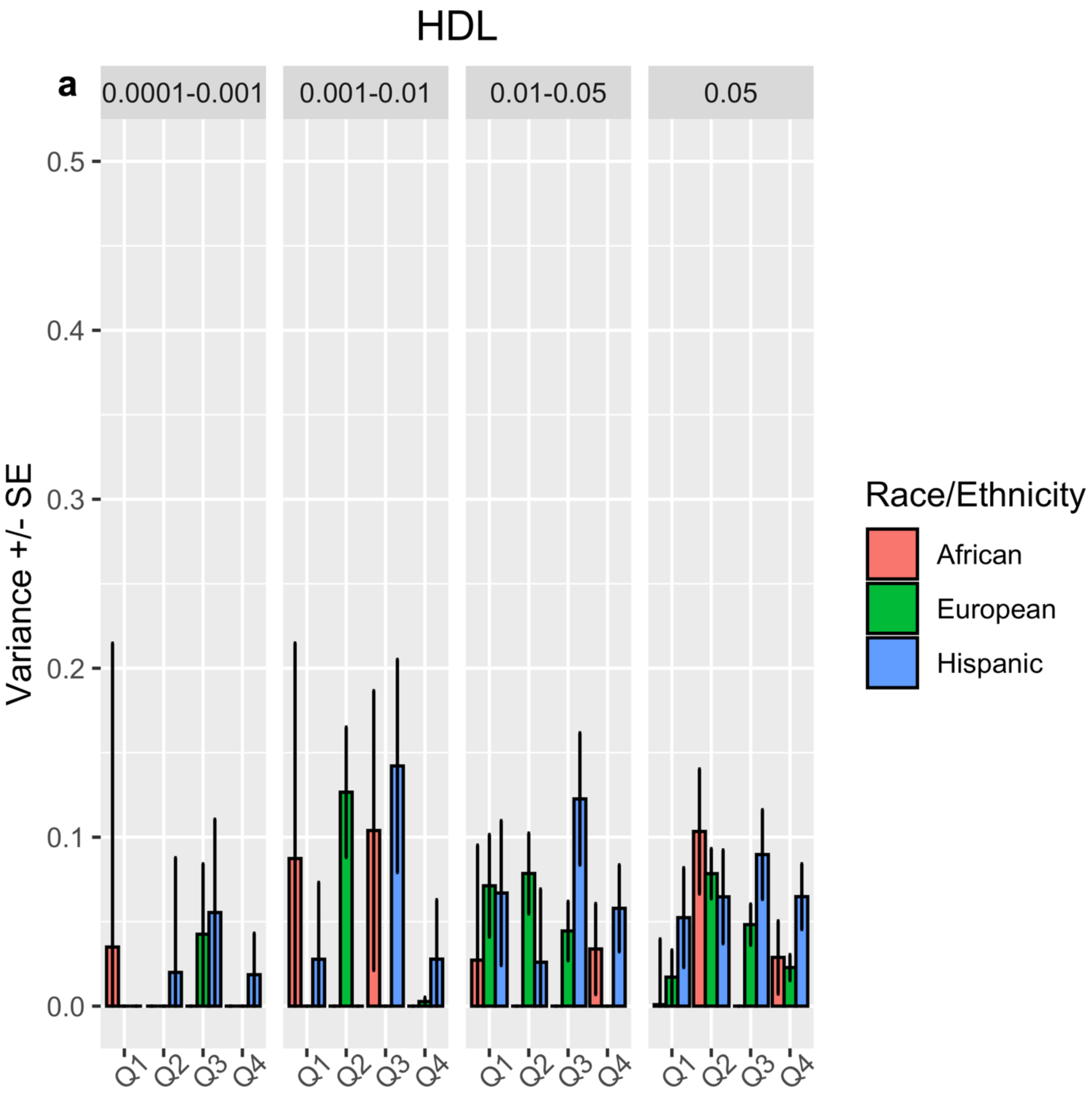
HDL
a
0.0001-0.001
0.001-0.01
0.01-0.05
0.05
Variance +/- SE
0.0
0.1
0.2
0.3
0.4
0.5
Q1
Q2
Q3
Q4
Race/Ethnicity
African
European
Hispanic

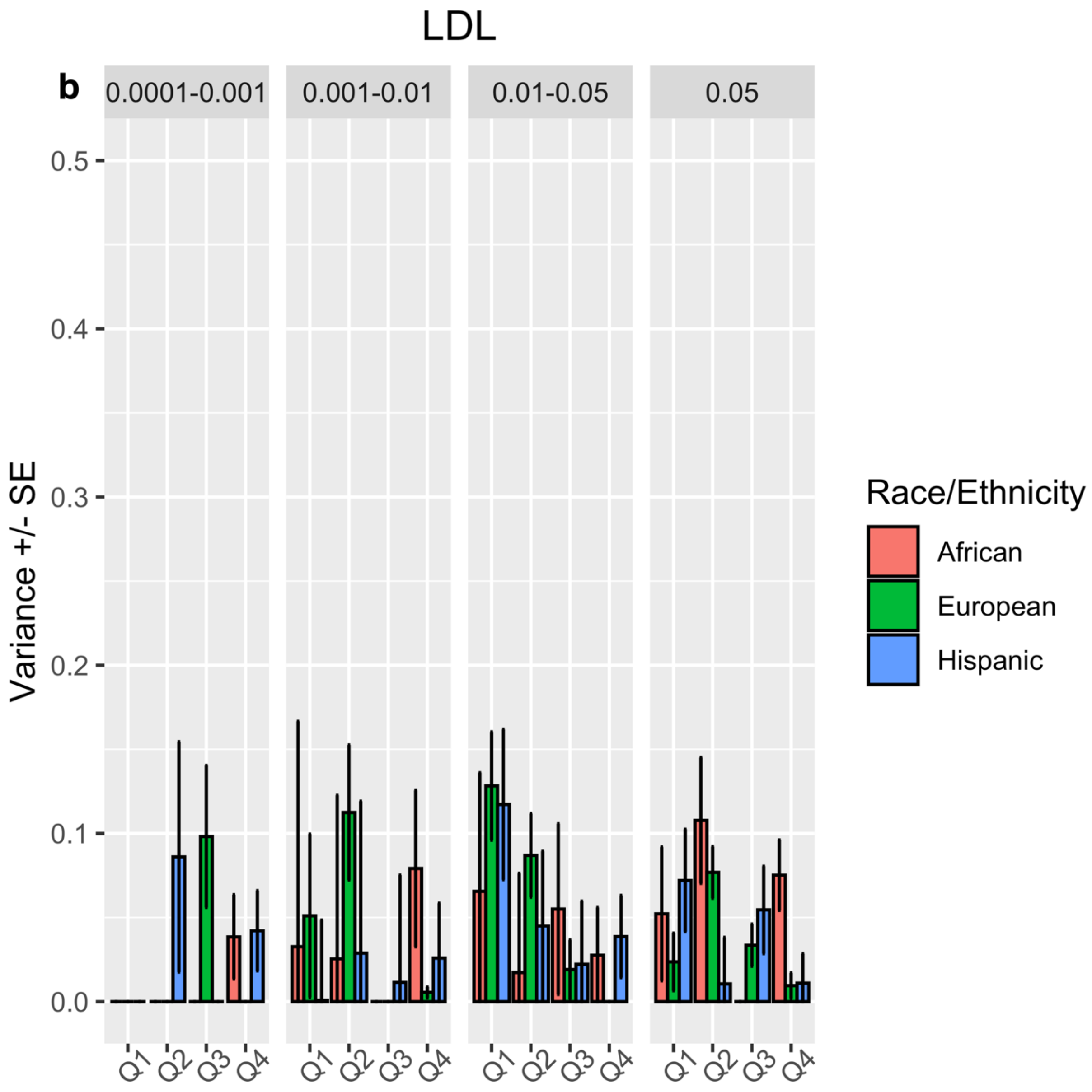

LDL
b
0.0001-0.001
0.001-0.01
0.01-0.05
0.05
Variance +/- SE
0.5
0.4
0.3
0.2
0.1
0.0
Q1
Q2
Q3
Q4
Race/Ethnicity
African
European
Hispanic

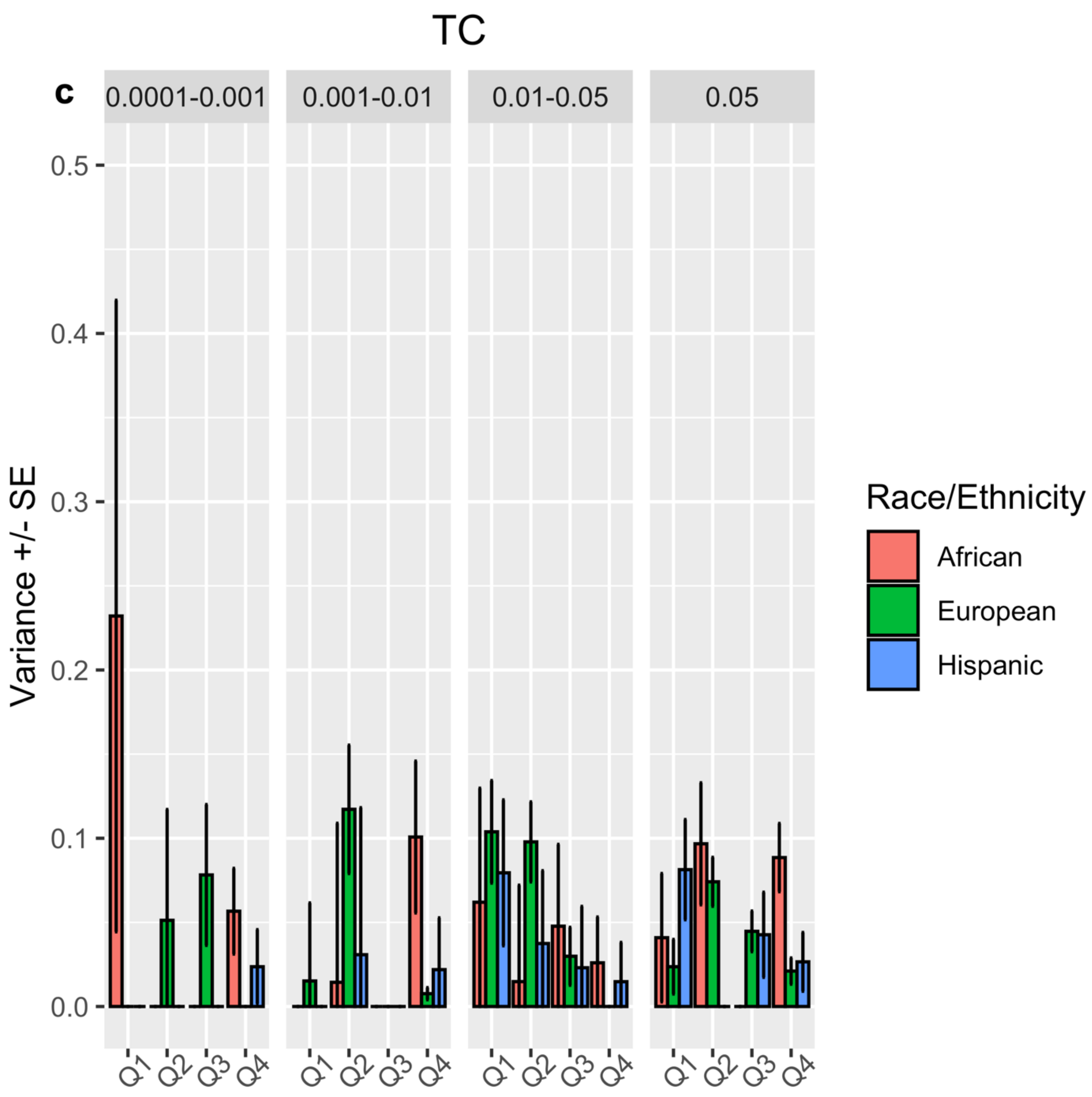

TC
c
0.0001-0.001
0.001-0.01
0.01-0.05
0.05
Variance +/- SE
0.0
0.1
0.2
0.3
0.4
0.5
Q1
Q2
Q3
Q4
Race/Ethnicity
African
European
Hispanic

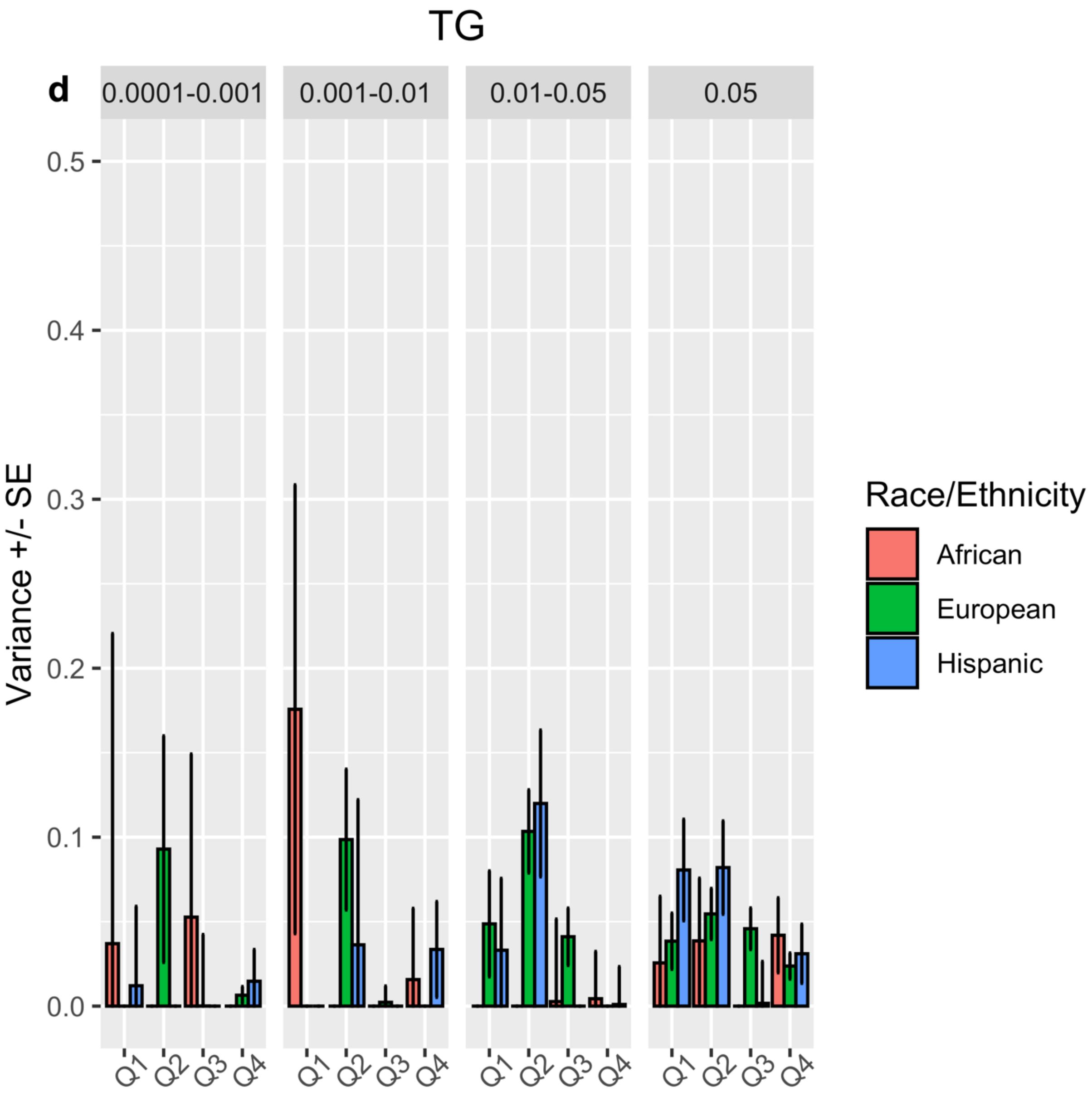

Supplementary Fig. 10

Distribution of heritability estimate (h^2) for three ancestral subgroups from TOPMed cohort. Heritability estimates were calculated using Greml-LDMS workflow for the four lipid phenotypes (HDL, LDL, TC, TG) from three ancestral groups (African, European, Hispanic). Variants were binned based on MAF cut-offs to 4 bins and were grouped to 4 quartiles based on LD scores. Any negative estimate was converted to zero and the h^2 values are plotted for each lipid. **a**, Heritability estimates for HDL-C. **b**, Heritability estimates for LDL-C. **c**, Heritability estimates for TC. **d**, Heritability estimates for TG.

HDL-C – High-Density Lipoprotein Cholesterol; LD – Linkage Disequilibrium; LDL-C – Low-Density Lipoprotein Cholesterol; MAF – Minor Allele Frequency; TC – Total Cholesterol; TG – Triglycerides.

Supplementary References:

1. Mitchell, B. D. *et al.* The genetic response to short-term interventions affecting cardiovascular function: rationale and design of the Heredity and Phenotype Intervention (HAPI) Heart Study. *Am. Heart J.* **155**, 823–828 (2008).
2. The Atherosclerosis Risk in Communities (ARIC) Study: design and objectives. The ARIC investigators. *Am. J. Epidemiol.* **129**, 687–702 (1989).
3. Nadkarni, G. N. *et al.* Apolipoprotein L1 Variants and Blood Pressure Traits in African Americans. *J. Am. Coll. Cardiol.* **69**, 1564–1574 (2017).
4. Hughes, G. H. *et al.* Recruitment in the Coronary Artery Disease Risk Development in Young Adults (Cardia) Study. *Control. Clin. Trials* **8**, 68S-73S (1987).
5. Redline, S. *et al.* The familial aggregation of obstructive sleep apnea. *Am. J. Respir. Crit. Care Med.* **151**, 682–687 (1995).
6. Fried, L. P. *et al.* The Cardiovascular Health Study: design and rationale. *Ann. Epidemiol.* **1**, 263–276 (1991).
7. Bowden, D. W. *et al.* Review of the Diabetes Heart Study (DHS) family of studies: a comprehensively examined sample for genetic and epidemiological studies of type 2 diabetes and its complications. *Rev. Diabet. Stud. RDS* **7**, 188–201 (2010).
8. Castelli, W. P. Epidemiology of coronary heart disease: the Framingham study. *Am. J. Med.* **76**, 4–12 (1984).
9. Kannel, W. B., Feinleib, M., McNamara, P. M., Garrison, R. J. & Castelli, W. P. An investigation of coronary heart disease in families. The Framingham offspring study. *Am. J. Epidemiol.* **110**, 281–290 (1979).
10. Kannel, W. B., Dawber, T. R., Kagan, A., Revotskie, N. & Stokes, J. Factors of risk in the development of coronary heart disease--six year follow-up experience. The Framingham Study. *Ann. Intern. Med.* **55**, 33–50 (1961).
11. Kannel, W. B., Castelli, W. P., Gordon, T. & McNamara, P. M. Serum cholesterol, lipoproteins, and the risk of coronary heart disease. The Framingham study. *Ann. Intern. Med.* **74**, 1–12 (1971).
12. Kral, B. G. *et al.* A common variant in the CDKN2B gene on chromosome 9p21 protects against coronary artery disease in Americans of African ancestry. *J. Hum. Genet.* **56**, 224–229 (2011).
13. Bray, P. F. *et al.* Heritability of platelet function in families with premature coronary artery disease. *J. Thromb. Haemost. JTH* **5**, 1617–1623 (2007).
14. FBPP Investigators. Multi-center genetic study of hypertension: The Family Blood Pressure Program (FBPP). *Hypertens. Dallas Tex 1979* **39**, 3–9 (2002).
15. Daniels, P. R. *et al.* Familial aggregation of hypertension treatment and control in the Genetic Epidemiology Network of Arteriopathy (GENOA) study. *Am. J. Med.* **116**, 676–681 (2004).
16. GenSalt Collaborative Research Group. GenSalt: rationale, design, methods and baseline characteristics of study participants. *J. Hum. Hypertens.* **21**, 639–646 (2007).
17. Higgins, M. *et al.* NHLBI Family Heart Study: objectives and design. *Am. J. Epidemiol.* **143**, 1219–1228 (1996).
18. Lavange, L. M. *et al.* Sample design and cohort selection in the Hispanic Community Health Study/Study of Latinos. *Ann. Epidemiol.* **20**, 642–649 (2010).

19. Sorlie, P. D. *et al.* Design and implementation of the Hispanic Community Health Study/Study of Latinos. *Ann. Epidemiol.* **20**, 629–641 (2010).
20. Arnett, D. K. *et al.* Sibling correlation of left ventricular mass and geometry in hypertensive African Americans and whites: the HyperGEN study. Hypertension Genetic Epidemiology Network. *Am. J. Hypertens.* **14**, 1226–1230 (2001).
21. Williams, R. R. *et al.* NHLBI family blood pressure program: methodology and recruitment in the HyperGEN network. Hypertension genetic epidemiology network. *Ann. Epidemiol.* **10**, 389–400 (2000).
22. Fuqua, S. R. *et al.* Recruiting African-American research participation in the Jackson Heart Study: methods, response rates, and sample description. *Ethn. Dis.* **15**, S6-18–29 (2005).
23. Wilson, J. G. *et al.* Study design for genetic analysis in the Jackson Heart Study. *Ethn. Dis.* **15**, S6-30–37 (2005).
24. Taylor, H. A. *et al.* Toward resolution of cardiovascular health disparities in African Americans: design and methods of the Jackson Heart Study. *Ethn. Dis.* **15**, S6-4–17 (2005).
25. Bild, D. E. *et al.* Multi-Ethnic Study of Atherosclerosis: objectives and design. *Am. J. Epidemiol.* **156**, 871–881 (2002).
26. Ellinor, P. T., Low, A. F., Patton, K. K., Shea, M. A. & Macrae, C. A. Discordant atrial natriuretic peptide and brain natriuretic peptide levels in lone atrial fibrillation. *J. Am. Coll. Cardiol.* **45**, 82–86 (2005).
27. Ellinor, P. T., Yoerger, D. M., Ruskin, J. N. & MacRae, C. A. Familial aggregation in lone atrial fibrillation. *Hum. Genet.* **118**, 179–184 (2005).
28. Mitchell, B. D. *et al.* Genetic and environmental contributions to cardiovascular risk factors in Mexican Americans. The San Antonio Family Heart Study. *Circulation* **94**, 2159–2170 (1996).
29. Hawley, N. L. *et al.* Prevalence of adiposity and associated cardiometabolic risk factors in the Samoan genome-wide association study. *Am. J. Hum. Biol. Off. J. Hum. Biol. Counc.* **26**, 491–501 (2014).
30. Minster, R. L. *et al.* A thrifty variant in CREBRF strongly influences body mass index in Samoans. *Nat. Genet.* **48**, 1049–1054 (2016).
31. Ranade, K. a genome scan for hypertension susceptibility loci in populations of Chinese and Japanese origins. *Am. J. Hypertens.* **16**, 158–162 (2003).
32. Wu, K.-D. *et al.* Clustering and heritability of insulin resistance in Chinese and Japanese hypertensive families: a Stanford-Asian Pacific Program in Hypertension and Insulin Resistance sibling study. *Hypertens. Res. Off. J. Jpn. Soc. Hypertens.* **25**, 529–536 (2002).
33. Manson, J. E. *et al.* Menopausal Hormone Therapy and Long-term All-Cause and Cause-Specific Mortality: The Women's Health Initiative Randomized Trials. *JAMA* **318**, 927–938 (2017).
34. Design of the Women's Health Initiative clinical trial and observational study. The Women's Health Initiative Study Group. *Control. Clin. Trials* **19**, 61–109 (1998).

Supplementary Note2:

The Amish studies were supported by NIH grants R01 AG18728, U01 HL072515, R01 HL088119, R01 HL121007, and P30 DK072488. The Atherosclerosis Risk in Communities (ARIC) study has been funded in whole or in part with Federal funds from the National Heart, Lung, and Blood Institute, National Institutes of Health, Department of Health and Human Services (contract numbers HHSN268201700001I, HHSN268201700002I, HHSN268201700003I, HHSN268201700004I and HHSN268201700005I). The authors thank the staff and participants of the ARIC study for their important contributions. The Mount Sinai BioMe Biobank (BioMe) has been supported by The Andrea and Charles Bronfman Philanthropies and in part by Federal funds from the NHLBI and NHGRI (U01HG00638001; U01HG007417; X01HL134588). Coronary Artery Risk Development in Young Adults (CARDIA) Study (phs001612) was performed at the Baylor College of Medicine Human genome Sequencing Center (contract HHSN268201600033I). Core support including centralized genomic read mapping and genotype calling, along with variant quality metrics and filtering were provided by the TOPMed Informatics Research Center (3R01HL-117626-02S1; contract HHSN268201800002I). Core support including phenotype harmonization, data management, sample-identity QC, and general program coordination were provided by the TOPMed Data Coordinating Center (R01HL-120393; U01HL-120393; contract HHSN268201800001I). We gratefully acknowledge the studies and participants who provided biological samples and data for TOPMed. The Coronary Artery Risk Development in Young Adults Study (CARDIA) is conducted and supported by the National Heart, Lung, and Blood Institute (NHLBI) in collaboration with the University of

Alabama at Birmingham (HHSN268201800005I & HHSN268201800007I), Northwestern University (HHSN268201800003I), University of Minnesota (HHSN268201800006I), and Kaiser Foundation Research Institute (HHSN268201800004I). Cleveland Family Study (CFS) is supported by grants from the NHLBI (HL046389, HL113338, and 1R35HL135818). Cardiovascular Health Study (CHS) was supported by contracts HHSN268201200036C, HHSN268200800007C, HHSN268201800001C, N01HC55222, N01HC85079, N01HC85080, N01HC85081, N01HC85082, N01HC85083, N01HC85086, 75N92021D00006, and grants U01HL080295 and U01HL130114 from the National Heart, Lung, and Blood Institute (NHLBI), with additional contribution from the National Institute of Neurological Disorders and Stroke (NINDS). Additional support was provided by R01AG023629 from the National Institute on Aging (NIA). A full list of principal CHS investigators and institutions can be found at CHS-NHLBI.org. The content is solely the responsibility of the authors and does not necessarily represent the official views of the National Institutes of Health. Diabetes Heart Study (DHS) was supported by HL92301, HL67348, NS058700, AR48797, DK071891, AG058921, the General Clinical Research Center of the Wake Forest University School of Medicine (RR07122, HL085989), the American Diabetes Association, and a pilot grant from the Claude Pepper Older Americans Independence Center of Wake Forest University Health Sciences (AG10484). Framingham Heart Study (FHS) acknowledges the support of contracts NO1-HC-25195 and HHSN268201500001I from the National Heart, Lung and Blood Institute and grant supplement R01 HL092577-06S1 for this research. WGS for "NHLBI TOPMed: Whole Genome Sequencing and Related Phenotypes in the Framingham Heart Study" (phs000974) was performed at the Broad Institute of MIT and

Harvard (HHSN268201500014C, 3R01HL092577-06S1, and 3U54HG003067-12S2). We also acknowledge the dedication of the FHS study participants without whom this research would not be possible. Genetic Studies of Atherosclerosis Risk (GeneSTAR) was supported by grants from the National Institutes of Health/National Heart, Lung, and Blood Institute (U01 HL72518, HL087698, HL49762, HL58625, HL071025, HL112064), the National Institutes of Health/National Institute of Nursing Research (NR0224103), and by a grant from the National Institutes of Health/National Center for Research Resources (M01-RR000052) to the Johns Hopkins General Clinical Research Center. Genetic Epidemiology Network of Arteriopathy (GENOA) was supported by the National Heart, Lung and Blood Institute (HL054457, HL054464, HL054481, HL087660, and HL119443) of the National Institutes of Health. Genetic Epidemiology Network of Salt Sensitivity (GenSalt) was supported by research grants (U01HL072507, R01HL087263, and R01HL090682) from the National Heart, Lung and Blood Institute, National Institutes of Health, Bethesda, MD. Genetics of Lipid-Lowering Drugs and Diet Network (GOLDN) biospecimens, baseline phenotype data, and intervention phenotype data were collected with funding from National Heart, Lung and Blood Institute (NHLBI) grant U01 HL072524. The Hispanic Community Health Study/Study of Latinos (HCHS-SOL) was carried out as a collaborative study supported by contracts from the National Heart, Lung, and Blood Institute (NHLBI) to the University of North Carolina (N01-HC65233), University of Miami (N01-HC65234), Albert Einstein College of Medicine (N01-HC65235), Northwestern University (N01-HC65236), and San Diego State University (N01-HC65237). The Hypertension Genetic Epidemiology Network and Genetic Epidemiology Network of Arteriopathy (HyperGEN) Study is part of the National

Heart, Lung, and Blood Institute (NHLBI) Family Blood Pressure Program; collection of the data represented here was supported by grants U01 HL054472 (MN Lab), U01 HL054473 (DCC), U01 HL054495 (AL FC), and U01 HL054509 (NC FC). The HyperGEN: Genetics of Left Ventricular Hypertrophy Study was supported by NHLBI grant R01 HL055673 with whole-genome sequencing made possible by supplement -18S1. The Jackson Heart Study (JHS) is supported and conducted in collaboration with Jackson State University (HHSN268201800013I), Tougaloo College (HHSN268201800014I), the Mississippi State Department of Health (HHSN268201800015I) and the University of Mississippi Medical Center (HHSN268201800010I, HHSN268201800011I and HHSN268201800012I) contracts from the National Heart, Lung, and Blood Institute (NHLBI) and the National Institute on Minority Health and Health Disparities (NIMHD). The authors also wish to thank the staffs and participants of the JHS. Multi-Ethnic Study of Atherosclerosis (MESA) and the MESA SHARe projects are conducted and supported by the National Heart, Lung, and Blood Institute (NHLBI) in collaboration with MESA investigators. Support for MESA is provided by contracts 75N92020D00001, HHSN268201500003I, N01-HC-95159, 75N92020D00005, N01-HC-95160, 75N92020D00002, N01-HC-95161, 75N92020D00003, N01-HC-95162, 75N92020D00006, N01-HC-95163, 75N92020D00004, N01-HC-95164, 75N92020D00007, N01-HC-95165, N01-HC-95166, N01-HC-95167, N01-HC-95168, N01-HC-95169, UL1-TR-000040, UL1-TR-001079, and UL1-TR-001420. Funding for SHARe genotyping was provided by NHLBI Contract N02-HL-64278. Genotyping was performed at Affymetrix (Santa Clara, California, USA) and the Broad Institute of Harvard and MIT (Boston, Massachusetts, USA) using the

Affymetrix Genome-Wide Human SNP Array 6.0. Also supported in part by NHLBI CHARGE Consortium Contract HL105756. The provision of genotyping data was supported in part by the National Center for Advancing Translational Sciences, CTSI grant UL1TR001881, and the National Institute of Diabetes and Digestive and Kidney Disease Diabetes Research Center (DRC) grant DK063491 to the Southern California Diabetes Endocrinology Research Center. Infrastructure for the CHARGE Consortium is supported in part by the National Heart, Lung, and Blood Institute (NHLBI) grant R01HL105756. The Massachusetts General Hospital Atrial Fibrillation Study (MGH-AF) was supported by grants to Dr. Ellinor from the Fondation Leducq (14CVD01), the National Institutes of Health to Dr. Ellinor (1RO1HL092577, R01HL128914, K24HL105780) and Dr. Lubitz (1R01HL139731) and by grants from the American Heart Association to Dr. Ellinor (18SFRN34110082) and to Dr. Lubitz (18SFRN34250007). San Antonio Family Study (SAFS) was supported in part by National Institutes of Health (NIH) grants R01 HL045522, MH078143, MH078111 and MH083824; and whole genome sequencing of SAFS subjects was supported by U01 DK085524 and R01 HL113323. We are very grateful to the participants of the San Antonio Family Study for their continued involvement in our research programs. Samoan Adiposity Study (SAS) was funded by NIH grant R01-HL093093. We thank the Samoan participants of the study and local village authorities. We acknowledge the support of the Samoan Ministry of Health and the Samoa Bureau of Statistics for their support of this research. The Rare Variants for Hypertension in Taiwan Chinese (THRV) is supported by the National Heart, Lung, and Blood Institute (NHLBI) grant (R01HL111249) and its participation in TOPMed is supported by an NHLBI supplement (R01HL111249-04S1). SAPPHIRe was

supported by NHLBI grants (U01HL54527, U01HL54498) and Taiwan funds, and the other cohorts were supported by Taiwan funds. The Women's Health Initiative (WHI) program is funded by the National Heart, Lung, and Blood Institute, National Institutes of Health, U.S. Department of Health and Human Services through contracts HHSN268201600018C, HHSN268201600001C, HHSN268201600002C, HHSN268201600003C, and HHSN268201600004C. The Centers for Common Disease Genomics (CCDG) program was supported by NHGRI and NHLBI, and whole genome sequencing was performed at the Baylor College of Medicine Human Genome Sequencing Center (UM1 HG008898 and R01HL059367).
