# Supplementary material for: Whole genome sequence analysis of blood lipid levels in >66,000 individuals
Source: Nat Commun. 2022 Oct 11;13:5995. doi: 10.1038/s41467-022-33510-7 (PMC9553944; doi:10.1038/s41467-022-33510-7)
Supplement: Supplementary file 3 — Description of Additional Supplementary Files [file 41467_2022_33510_MOESM3_ESM.pdf]

## Description of Additional Supplementary Files

File Name: Supplementary Data 1
Description: **TOPMed freeze 8 phenotype data distributed based on 21 cohorts**. The Freeze8 lipid data is composed of 66329 samples. Samples are tabulated based on gender, ancestral groups, sequencing centers and lipid treatment (percentage of samples) for each cohort. Distribution of mean (standard deviation) for sex, unadjusted non-transformed lipid concentration of HDL-C, LDL-C, TC and TG in full sample group and stratified groups based on gender and ancestry are provided. Since TG has a skewed distribution, the median and IQR for TG concentration are provided separately for all samples. Lipid concentrations are in units of mg/dl. Cohorts with no values for any specific columns are represented as NA. Sequencing centers: Baylor: Baylor College of Medicine Human Genome Sequencing Center, Broad: Broad Institute of MIT and Harvard, Illumina: Illumina Genomic Services, Macrogen: PSOMAGEN (formerly Macrogen), NYGC: New York Genome Center, UW: McDonnell Genome Institute (MGI) at Washington University, WASHU: Northwest Genomics Center. HDL-C – High-Density Lipoprotein Cholesterol; LDL-C – Low-Density Lipoprotein Cholesterol; TC – Total Cholesterol; TG – Triglycerides

File Name: Supplementary Data 2
Description: **TOPMed Freeze 8 genotype distribution based on chromosome.** Variant counts by chromosome are tabulated across MAF and MAC bins. MAF – Minor Allele Frequency; MAC – Minor Allele Count

File Name: Supplementary Data 3
Description: **Significant genomic loci idented by FUMA.** For each lipid, significant loci identified by SNP2GENE function is documented along with the independent significant SNPS

File Name: Supplementary Data 4
Description: **TOPMed Freeze 8 variants summary.** Variants which passed the significance criteria were clumped (window 250 kb, $r^2$ 0.5) and compared against MVP summary statistic and GWAS catalog. Variants were binned to three categories, Known-Position (variant previously associated), Known-Loci (variants not previously significantly associated with the corresponding lipid phenotype but within 500 kb of a known locus) and Novel. The list of variants is tabulated for each lipid phenotype and each category of is ordered based on chromosome position. Summary statistics reported were obtained from two-sided genetic association testing preformed using SAIGE-QT model, where the model was adjusted for all the covariates. TOPMed – Trans-Omics for Precision Medicine; MVP – Million Veteran Program; GWAS – Genome Wide Association Study.

File Name: Supplementary Data 5
Description: **Ancestry specificity and replication of putative novel variants.** List of novel single variants identified after comparing with MVP summary stats are tabulated. Effects, p-values MAF of variants in discovery (TOPMed) cohort specific to each ancestral group is documented**.** Effects and p-values from replication cohort (MGB and Penn biobank) are documented. All effect estimates are in mg/dL units, except for TG which was log-transformed in analysis thereby representing fractional change. Summary statistics reported were obtained from two-sided genetic association testing preformed using SAIGE-QT model for the discovery cohort and linear regression model for the replication cohorts, where the models were adjusted for all the covariates. MVP – Million Veteran Program; MAF – Minor Allele Frequency; TOPMed – Trans-Omics for Precision Medicine; MGB – Mass General Brigham.

File Name: Supplementary Data 6
Description: **Baseline characteristics of replication cohorts.** Sample sizes, gender distributions, ancestry distributions, and mean ages of individuals in each of the replication cohort are provided. Mean (standard deviation) of unadjusted and nontransformed lipid concentrations are presented for the MGB Biobank and Penn Medicine Biobank.
MGB – Mass General Brigham.

File Name: Supplementary Data 7
Description: **Evaluation of suggestive lipid alleles in TOPMed with independent datasets.** Putative novel variants at 'suggestive' p-values ($5x10^{-07}$ – $5x10^{-09}$) in TOPMed are listed, including ancestry-specific effects. Evidence for association in the MGB and Penn Medicine Biobanks are also included. Summary statistics reported were obtained from two-sided genetic association testing preformed using SAIGE-QT model for the discovery cohort and linear regression model for the replication cohorts, where the models were adjusted for all the covariates. TOPMed – Trans-Omics for Precision Medicine; MGB – Mass General Brigham.

File Name: Supplementary Data 8
Description: **List of differentially expressed genes**. Top 5 up-regulated and down-regulated genes identified in FUMA eQTL enrichment analysis are documented.
eQTL – Expression Quantitative Trait Loci

File Name: Supplementary Data 9
Description: **List of enriched pathways and gene sets.** Significantly enriched gene sets from MsigDB and pathways from different databases from FUMA analysis are documented.

File Name: Supplementary Data 10
Description: **List of *CETP* variants significant at suggestive p-value ($5X10^{-07}$) from both LDL-C and HDL-C GWAS**. All the variants were highly significant to HDL-C and the White ancestry group contributes mostly for the association with positive effects. Three variants are genome significant ($5X10^{-09}$) and two of them are suggestive significant with LDL-C association. The Black ancestry group contributed to strong LDL-C association with negative effects. Effects, p-values and MAF of trans-ancestry and ancestry-specific associations are documented. Summary statistics reported were obtained from two-sided genetic association testing preformed using SAIGE-QT model, where the model was adjusted for all the covariates. HDL-C – High-Density Lipoprotein Cholesterol; LDL-C – Low-Density Lipoprotein Cholesterol; GWAS – Genome Wide Association Studies; MAF – Minor Allele Frequency.

File Name: Supplementary Data 11
Description: **Phenome wide association results.** PheWAS with UKB disease data was carried out for the three replicated single variants. Significant complex traits with FDR < 0.05 is documented. Summary statistics reported were obtained from two-sided linear regression model, where the model was adjusted for all the covariates. FDR – False Discovery Rate; UKB – UK Biobank.

File Name: Supplementary Data 12
Description: **Significant gene-centric coding rare variant aggregate sets.** Each of the significant (p-value < $2.5x10^{-06}$) rare (MAF<1%) coding aggregate sets for at least one lipid phenotype are listed by gene name and mask. Aggregates are ordered by mask and number of variants in each set is documented. SKAT, Burden, ACAT-O and STAAR-O p-values for each of the aggregate sets are provided. Summary statistics reported were obtained from two-sided aggregate testing preformed using STAAR gene-centric model, where the model was adjusted for all the covariates. MAF – Minor Allele Frequency; SKAT – SNP-set (Sequence) Kernel Association Test; ACAT – Aggregated Cauchy Association Test; STAAR – variant-Set Test for Association using Annotation information.

File Name: Supplementary Data 13
Description: **Significant gene-centric non-coding rare variant aggregate sets.** Significant (p-value < $2.5x10^{-06}$) rare (MAF<1%) non-coding aggregate sets for at least one lipid phenotype are listed. Aggregates are ordered by masks and the number of variants in each aggregate set is documented. SKAT, Burden, ACAT-O and STAAR-O p-values for each of the aggregate sets are provided. Summary statistics reported were obtained from two-sided aggregate testing preformed using STAAR gene-centric model, where the model was adjusted for all the covariates. MAF – Minor Allele Frequency; SKAT – SNP-set (Sequence) Kernel Association Test; ACAT – Aggregated Cauchy Association Test; STAAR – variant-Set Test for Association using Annotation information.

File Name: Supplementary Data 14
Description: **Significant gene-centric coding rare variant aggregate sets after conditional analysis.** Each of the rare coding aggregate sets significant genome wide (after conditional analysis on known common variants) are listed for each lipid phenotypes. The list of common variants that were used for adjustment is provided with RS ids. Aggregates are ordered based on STAAR-O p-values and the number of variants in each aggregate set is documented. SKAT, Burden, ACAT-O and STAAR-O p-values for each of the aggregate sets are provided. The effect estimates and corresponding p-values from Glmm.Wald test is documented. Results from UK Biobank replication is documented with number of variants tested and STAAR-O p-values. Summary statistics reported were obtained from two-sided aggregate testing preformed using STAAR gene-centric model and Glmm.Wald test, where the model was adjusted for all the covariates. SKAT – SNP-set (Sequence) Kernel Association Test; ACAT – Aggregated Cauchy Association Test; STAAR – variant-Set Test for Association using Annotation information.

File Name: Supplementary Data 15
Description: **Significant gene-centric non-coding rare variant aggregate sets after conditional analysis.** Each of the rare non-coding aggregate sets significant genome wide (after conditional analysis on known common variants) are listed for each lipid phenotypes. The list of common variants that were used for adjustment is provided with RS ids. Aggregates are ordered based on STAAR-O p-values and the number of variants in each aggregate set is documented. SKAT, Burden, ACAT-O and STAAR-O p-values for each of the aggregate sets are provided. The effect estimates and corresponding p-values from Glmm.Wald test is documented. Results from UK Biobank replication is documented with number of variants tested and STAAR-O p-values. Summary statistics reported were obtained from two-sided aggregate testing preformed using STAAR gene-centric model and Glmm.Wald test, where the models were adjusted for all the covariates. SKAT – SNP-set (Sequence) Kernel Association Test; ACAT – Aggregated Cauchy Association Test; STAAR – variant-Set Test for Association using Annotation information.

File Name: Supplementary Data 16
Description: **Region-based sliding-window results before and after conditional analysis.** Regions that are significantly associated with at least one lipid phenotype after conditioning on significant single variants are listed. The start and end location of the region, the number of variants comprising the aggregate sets and the list of variants which were conditioned are provided. Aggregates are ordered based on STAAR-O p-values and the number of variants in each aggregate set is documented. SKAT, Burden, ACAT and STAAR-O p-values before and after conditional analysis for each of the aggregate sets are provided. Genes mapped to the region and the intron-exon boundaries are provided. Glmm.Wald test was implemented on all the variants of the aggregate set and only non-coding variants for each aggregate. The corresponding effect estimates, and p-values is documented. Results from UK Biobank replication is documented with number of variants tested and STAAR-O p-values. Summary statistics reported were obtained from two-sided aggregate testing preformed using STAAR sliding-window model and Glmm.Wald test, where the models were adjusted for all the covariates. SKAT – SNP-set (Sequence)

Kernel Association Test; ACAT – Aggregated Cauchy Association Test; STAAR – variant-Set Test for Association using Annotation information.

File Name: Supplementary Data 17
Description: **Region-based dynamic-window results before and after conditional analysis.** Regions that are significantly associated with at least one lipid phenotype by SCANG after conditioning on significant single variants are listed. The start and end location of the region, the number of variants comprising the aggregate sets and the list of variants which were conditioned are provided. Aggregates are ordered based on STAAR-O p-values and the number of variants in each aggregate set is documented. SKAT, Burden, ACAT and STAAR-O p-values before and after conditional analysis for each of the aggregate sets are provided. Unconditional p-values are obtained from each of the test where the aggregate set is significant, and the conditional p-values are summarized for each aggregate set. Genes mapped to the regions and the intron-exon boundaries are provided. Glmm.Wald test was implemented on all the variants of the aggregate set and only non-coding variants for each aggregate. The corresponding effect estimates, and p-values is documented. Results from UK Biobank replication is documented with number of variants tested and STAAR-O p-values. Summary statistics reported were obtained from two-sided aggregate testing preformed using STAAR dynamic-window model and Glmm.Wald test, where the models were adjusted for all the covariates. SKAT – SNP-set (Sequence) Kernel Association Test; ACAT – Aggregated Cauchy Association Test; STAAR – variant-Set Test for Association using Annotation information.

File Name: Supplementary Data 18
Description: **Significant rare non-coding variants after conditioning on rare coding variants.** Rare non-coding aggregates sets were additionally adjusted for rare coding variants of the same gene and the nearby gene. The nearby gene pairs are as follows: *LDLR-SPC24*, *CETP-HERPUD1*, *APOC3-APOA1*. STAAR-O p-values at each adjustment step is provided and aggregates tested with nearby gene is additionally shown. Summary statistics reported were obtained from two-sided aggregate testing preformed using STAAR gene-centric model, where the model was adjusted for all the covariates.

File Name: Supplementary Data 19
Description: **Common variants and rare variant aggregates at Mendelian lipid genes.** Twenty-two Mendelian lipid genes and their common variant and rare variant aggregates from coding and non-coding tests are listed. Summary statistics reported were obtained from two-sided aggregate testing preformed using STAAR gene-centric model, where the model was adjusted for all the covariates.

File Name: Supplementary Data 20
Description: **Heritability estimates from Greml-LDMS workflow.** Heritability estimates ($h^2$) were calculated for LD pruned variants from unrelated individuals from three ancestral groups (African, European and Hispanic). Variants were binned based on MAF and grouped into 4 quartiles based on LD score. $h^2$ values were calculated for each bin and any negative value was made zero. Total $h^2$ was calculated for each ancestry and lipids. Similar workflow was implemented with a set of variants from MGB Biobank array-genotypes and $h^2$ was calculated for each ancestry and lipids. LD – Linkage Disequilibrium; MAF – Minor Allele Frequency; MGB – Mass General Brigham.
