# Supplementary material for: Whole genome sequence analysis of blood lipid levels in >66,000 individuals
Source: Nat Commun. 2022 Oct 11;13:5995. doi: 10.1038/s41467-022-33510-7 (PMC9553944; doi:10.1038/s41467-022-33510-7)
Supplement: Supplementary file 5 — Reporting Summary [file 41467_2022_33510_MOESM5_ESM.pdf]

Corresponding author(s): Pradeep Natarajan

Last updated by author(s): Aug 19, 2022

Reporting Summary

Nature Portfolio wishes to improve the reproducibility of the work that we publish. This form provides structure for consistency and transparency in reporting. For further information on Nature Portfolio policies, see our Editorial Policies and the Editorial Policy Checklist.

Statistics

For all statistical analyses, confirm that the following items are present in the figure legend, table legend, main text, or Methods section.

n/a	Confirmed	
☐	☒	The exact sample size (*n*) for each experimental group/condition, given as a discrete number and unit of measurement
☐	☒	A statement on whether measurements were taken from distinct samples or whether the same sample was measured repeatedly
☐	☒	The statistical test(s) used AND whether they are one- or two-sided<br>*Only common tests should be described solely by name; describe more complex techniques in the Methods section.*
☐	☒	A description of all covariates tested
☐	☒	A description of any assumptions or corrections, such as tests of normality and adjustment for multiple comparisons
☐	☒	A full description of the statistical parameters including central tendency (e.g. means) or other basic estimates (e.g. regression coefficient) AND variation (e.g. standard deviation) or associated estimates of uncertainty (e.g. confidence intervals)
☐	☒	For null hypothesis testing, the test statistic (e.g. *F*, *t*, *r*) with confidence intervals, effect sizes, degrees of freedom and *P* value noted<br>*Give P values as exact values whenever suitable.*
☒	☐	For Bayesian analysis, information on the choice of priors and Markov chain Monte Carlo settings
☒	☐	For hierarchical and complex designs, identification of the appropriate level for tests and full reporting of outcomes
☐	☒	Estimates of effect sizes (e.g. Cohen's *d*, Pearson's *r*), indicating how they were calculated

Our web collection on statistics for biologists contains articles on many of the points above.

Software and code

Policy information about availability of computer code

Data collection	TOPMed Freeze8 Whole Genome Sequenced (WGS) data; UK Biobank (UKB) WGS data; Mass General Brigham (MGB) Biobank array-genotype data; Penn Medicine Biobank array-genotype data; UKB Imputed data.
Data analysis	Codes used to implement STAAR workflows are available at https://github.com/xihaoli/STAAR and https://github.com/xihaoli/STAARpipeline. (version 3.6.1)<br>Workflow implemented for whole genome heritability calculations are available at https://github.com/CNSGenomics/Heritability_WGS.<br>Other softwares: IMPUTE 4 program (https://jmarchini.org/software/); EAGLE (Version 2.3.4); Minimac (Version1); MAGMA (Version 1.08)

For manuscripts utilizing custom algorithms or software that are central to the research but not yet described in published literature, software must be made available to editors and reviewers. We strongly encourage code deposition in a community repository (e.g. GitHub). See the Nature Portfolio guidelines for submitting code & software for further information.

Data

Policy information about availability of data

All manuscripts must include a data availability statement. This statement should provide the following information, where applicable:

- Accession codes, unique identifiers, or web links for publicly available datasets
- A description of any restrictions on data availability
- For clinical datasets or third party data, please ensure that the statement adheres to our policy

Individual whole-genome sequence data for TOPMed and harmonized lipids at individual sample level are available through restricted access via the TOPMed dbGAP Exchange area. Summary level genotype data from TOPMed are available through the BRAVO browser (https://bravo.sph.umich.edu/). The UK Biobank (UKB) whole-genome sequence data can be accessed through UKB Research Analysis Platform (RAP), through the UKB approval system (https://www.ukbiobank.ac.uk). The Mass General Brigham Biobank (MGBB) individual-level data are available from https://personalizedmedicine.partners.org/Biobank/Default.aspx, where the data is available through institutional review board (IRB) approval, therefore not publicly available. Individual-level from Penn Medicine BioBank is not publicly available due to research participants privacy concerns. The summary data captured using whole exome sequencing can be accessed though PMBB Genome Browser (https://pmbb.med.upenn.edu/allele-frequency/). The dbGaP accessions for TOPMed cohorts are as follows: Old Order Amish (Amish) phs000956 and phs00039; Atherosclerosis Risk in Communities study (ARIC) phs001211 and phs000280; Mt Sinai BioMe Biobank (BioMe) phs001644 and phs000925; Coronary Artery Risk Development in Young Adults (CARDIA) phs001612 and phs000285; Cleveland Family Study (CFS) phs000954 and phs000284; Cardiovascular Health Study (CHS) phs001368 and phs000287; Diabetes Heart Study (DHS) phs001412 and phs001012; Framingham Heart Study (FHS) phs000974 and phs000007; Genetic Studies of Atherosclerosis Risk (GeneSTAR) phs001218 and phs000375; Genetic Epidemiology Network of Arteriopathy (GENOA) phs001345 and phs001238; Genetic Epidemiology Network of Salt Sensitivity (GenSalt) phs001217 and phs000784; Genetics of Lipid-Lowering Drugs and Diet Network (GOLDN) phs001359 and phs000741; Hispanic Community Health Study - Study of Latinos (HCHS_SOL) phs001395 and phs000810; Hypertension Genetic Epidemiology Network and Genetic Epidemiology Network of Arteriopathy (HyperGEN) phs001293 and phs001293; Jackson Heart Study (JHS) phs000964 and phs000286; Multi-Ethnic Study of Atherosclerosis (MESA) phs001416 and phs000209; Massachusetts General Hospital Atrial Fibrillation Study (MGH_AF) phs001062 and phs001001; San Antonio Family Study (SAFS) phs001215 and phs000462; Samoan Adiposity Study (SAS) phs000972 and phs000914; Taiwan Study of Hypertension using Rare Variants (THRV) phs001387 and phs001387; Women's Health Initiative (WHI) phs001237 and phs000200.

Human research participants

Policy information about studies involving human research participants and Sex and Gender in Research.

Reporting on sex and gender	We did not carry out sex-specific analysis. Sex was a covariate in our analysis.
Population characteristics	Multi-ancestral cohort from TOPMed was used in this study. Self reported race was used as covariate in the models.
Recruitment	Each cohort recruited participants, details are provided in the supplementary text.
Ethics oversight	TOPMed, Massachusetts General Hospital.

Note that full information on the approval of the study protocol must also be provided in the manuscript.

Field-specific reporting

Please select the one below that is the best fit for your research. If you are not sure, read the appropriate sections before making your selection.

☒ Life sciences ☐ Behavioural & social sciences ☐ Ecological, evolutionary & environmental sciences

For a reference copy of the document with all sections, see nature.com/documents/nr-reporting-summary-flat.pdf

Life sciences study design

All studies must disclose on these points even when the disclosure is negative.

Sample size	This study is conducted primarily to investigate plasma lipids from whole genome sequenced TOPMed dataset. We included all the samples with plasma lipid measure (N=66329). We did not carry out independent sample-size calcualtions in this study and choose to include all 66329 samples to maximize the cohort size.
Data exclusions	All samples and variants passing the central QC were included in this study.
Replication	For our single variant GWAS novel variants (N=7), we carried out replication using array-genotype imputed datasets from MGBB, Penn BioBank and UK Biobank data. For our rare variant GWAS aggregate sets, we replicated in UKB WGS data. The number of replicated aggregate sets with the corresponding p-values are provided in the supplementary tables. All replication attempts were successful.
Randomization	No randomization was performed. Since this is a population based study and did not focus on a treatment effect, randomization was not performed.

Blinding	No blinding was performed. Blinding was not performed as there was no randomization. Investigators did not have any access to identifying information.

Reporting for specific materials, systems and methods

We require information from authors about some types of materials, experimental systems and methods used in many studies. Here, indicate whether each material, system or method listed is relevant to your study. If you are not sure if a list item applies to your research, read the appropriate section before selecting a response.

Materials & experimental systems

n/a	Involved in the study
☒	☐ Antibodies
☒	☐ Eukaryotic cell lines
☒	☐ Palaeontology and archaeology
☒	☐ Animals and other organisms
☒	☐ Clinical data
☒	☐ Dual use research of concern

Methods

n/a	Involved in the study
☒	☐ ChIP-seq
☒	☐ Flow cytometry
☒	☐ MRI-based neuroimaging
